# The Lyn/RUVBL1 Complex Promotes Colorectal Cancer Liver Metastasis by Regulating Arachidonic Acid Metabolism Through Chromatin Remodeling

**DOI:** 10.1002/advs.202406562

**Published:** 2024-12-12

**Authors:** Zhenyu Zhang, Yina Gao, Yuanyuan Qian, Bowen Wei, Kexin Jiang, Zhiwei Sun, Feifan Zhang, Mingming Yang, Salem Baldi, Xiaoqi Yu, Yunfei Zuo, Shuangyi Ren

**Affiliations:** ^1^ Department of General Surgery The Second Hospital of Dalian Medical University Dalian 116023 China; ^2^ Department of Clinical Biochemistry College of Laoratory Medicine, Dalian Medical University Dalian 116044 China

**Keywords:** arachidonic acid metabolism, chromatin remodeling, colorectal cancer liver metastasis, Lyn, RUVBL1

## Abstract

Liver metastasis is a common cause of death in colorectal cancer (CRC) patients, but epigenetic remodeling and metabolic reprogramming for CRC liver metastasis remain unclear. The study revealed that the Lyn/RUVBL1 complex is highly expressed in CRC and is closely correlated with liver metastasis. On the one hand, ATAC‐seq and HiCut suggested that Lyn/RUVBL1 regulates the expression of TRIB3 through the POL II‐mediated chromatin conformation of TRIB3 and thus the expression of β‐catenin. This promotes the proliferation and migration of CRC through β‐catenin‐mediated upregulation of MMP9 and VEGF. On the other hand, metabolomics revealed that Lyn/RUVBL1 regulates the expression of PGE2 through the enzyme COX2, thereby promoting arachidonic acid (AA) metabolism. CUT‐Tag showed that Lyn/RUVBL1 silencing reduces the H3K27ac level in the COX2 promoter. Then, it is found that COX2 is regulated by the transcription factor FOXA1. Lyn/RUVBL1 modulates AA metabolism by regulating the chromatin accessibility of FOXA1. AA metabolism promotes the metastasis of CRC by affecting β‐catenin nuclear translocation and upregulating MMP9 and VEGF. These findings suggest that the Lyn/RUVBL1 complex mediates epigenetic remodeling to regulate the metabolic reprogramming of AA, highlighting its role in promoting the metastasis of CRC.

## Graphical abstract

This study identified a noval complex Lyn/RUVBL1 that facilitates chromatin remodeling and activates TRIB3, which in turn drives β‐catenin to promote CRC metastasis. Furthermore, the study reveals that the Lyn/RUVBL1 complex regulates the arachidonic acid metabolism through up‐regulating of COX2 mediated by the transcription factor FOXA1. The findings suggested the Lyn/RUVBL1 complex mediates epigenetic remodeling to regulate AA metabolism.

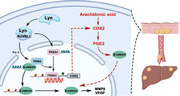

## 1 Introduction

CRC is the second leading cause of cancer‐related death.^[^ ^1^ ^]^ Liver metastasis is the predominant cause of mortality in CRC patients, and the 5‐year survival rate for patients with distant metastases is 14%.^[^ ^2^, ^3^ ^]^ Therefore, comprehensive research on the key regulatory targets of liver metastasis in CRC is crucial for advancing the precision treatment of this disease.

Epigenetic processes include chromatin remodeling, histone modifications, DNA methylation, and noncoding RNA expression.^[^ ^4^ ^]^ Spatially resolved multiomics analysis of primary CRC has revealed the occurrence of chromatin‐modifying gene DNA mutations and somatic chromatin remodeling in CRC.^[^ ^5^ ^]^ During tumorigenesis, there is an increase in the genome‐wide binding accessibility of transcription factors. The process of colorectal carcinogenesis involves DNA methylation changes in sporadic CRC that are closely associated with HNF4A motif‐related accessibility changes in the epithelium.^[^ ^6^ ^]^ Studies have revealed that decreased levels of H4K20me3 are linked to increased chromatin remodeling and R‐loop formation. The lysine methyltransferase SUV420H2‐mediated H4K20me3 is crucial for maintaining chromatin compaction, and its loss can drive the progression and metastasis of CRC.^[^ ^7^ ^]^ Increasing evidence suggests a close relationship between liver metastasis of CRC and chromatin remodeling.

CRC is a metabolic disease caused by a series of genetic alterations.^[^ ^8^ ^]^ The process of metabolic reprogramming enables cells to increase their survival and growth by modifying metabolic pathways to meet energy requirements.^[^ ^9^ ^]^ Metabolic fingerprinting indicates that the carcinogenic phosphoinositide‐3‐kinase regulatory subunit 1 can lead to the upregulation of arachidonic acid and its derivatives, thereby promoting tumor cell proliferation.^[^ ^10^ ^]^ Solute carrier family 25 member 1 promotes lipid synthesis to facilitate CRC growth.^[^ ^11^ ^]^ Aldolase B ‐mediated fructose metabolism drives metabolic reprogramming in the liver metastasis of colon cancer.^[^ ^12^ ^]^ These studies suggest that metabolic reprogramming plays an important role in CRC liver metastasis.

Epigenetic remodeling and metabolic reprogramming are two hallmark features of tumors.^[^ ^13^ ^]^ The MORF4‐related gene on chromosome 15 controls the transcriptional activity of lipid metabolism genes by regulating chromatin accessibility.^[^ ^14^ ^]^ Lysine demethylase 3A is responsible for reducing H3K9me2 modification in the promoter region of the phosphoglycerate kinase 1 gene, thereby promoting glycolysis and tumor progression in bladder cancer.^[^ ^15^ ^]^ The distant metastasis of pancreatic ductal adenocarcinoma involves the participation of chromatin remodeling and reprogramming of the oxidative pentose phosphate pathway.^[^ ^16^ ^]^ However, there has been limited research on the role of epigenetic remodeling and metabolic reprogramming in the liver metastasis of CRC.

Three back‐to‐back papers published in *Science* revealed a map of interactions between tumor proteins in 2021.^[^ ^17^, ^18^, ^19^ ^]^ Protein–protein interactions (PPIs) are crucial for tumor cell invasion and play a significant role in regulating tumor cell epigenetic remodeling, metabolic reprogramming, DNA repair, and ribonucleoprotein assembly.^[^ ^20^ ^]^ Protein arginine methyltransferase 1 (PRMT1)‐mediated H4R3me2a facilitates the recruitment of SMARCA4 and PRMT1 to activate downstream epidermal growth factor receptor and tensin 4 transcription, promoting the proliferation and migration of CRC.^[^ ^21^ ^]^ Bromodomain‐containing protein 4 phosphorylation induces chromatin remodeling in CRC by enhancing the interaction with the signal transducer and activator of transcription 3 through the simultaneous binding of enhancers and super‐enhancers, thereby facilitating tumor transcriptional programs.^[^ ^22^ ^]^ Lck/yes‐related protein tyrosine kinase (Lyn), a member of the Src family of kinases (SFKs), is involved in regulating cell proliferation, differentiation, apoptosis, migration, metabolism, and the immune response.^[^ ^23^ ^]^ Lyn forms a complex with EPH receptor A2 (EPHA2), activates Lyn, and phosphorylates TWIST1 to promote breast cancer invasion and metastasis.^[^ ^24^ ^]^ Lyn interacts with CD24 to activate ERK1/2 and promote the metastasis of CRC.^[^ ^25^ ^]^ However, the role of the Lyn complex in chromatin remodeling and metabolic reprogramming is unclear.

In this study, we discovered that a novel Lyn/RUVBL1 complex plays a role in promoting liver metastasis in CRC. We determined that Lyn/RUVBL1 facilitates chromatin remodeling through RNA polymerase II (POL II) and activates tribbles homolog 3 (TRIB3) to drive β‐catenin via epigenetic techniques such as ATAC‐seq and HiCut. Additionally, the Lyn/RUVBL1 complex has been found to regulate AA metabolism through forkhead box protein A1 (FOXA1) via metabolomics. Our findings establish a link between epigenetic remodeling and metabolic reprogramming. This study demonstrated that the Lyn/RUVBL1 complex regulates AA metabolism through epigenetic remodeling, leading to β‐catenin nuclear translocation, the upregulation of MMP9 and VEGF expression, and the promotion of CRC liver metastasis. Understanding the role and mechanism of this complex in promoting CRC liver metastasis will increase our understanding of the involvement of epigenetic remodeling and metabolic reprogramming in CRC liver metastasis.

## 2 Results

### 2.1 The Lyn and RUVBL1‐Forming Complex is Highly Expressed in CRC and is Closely Associated with Liver Metastasis

**Figure 1:**
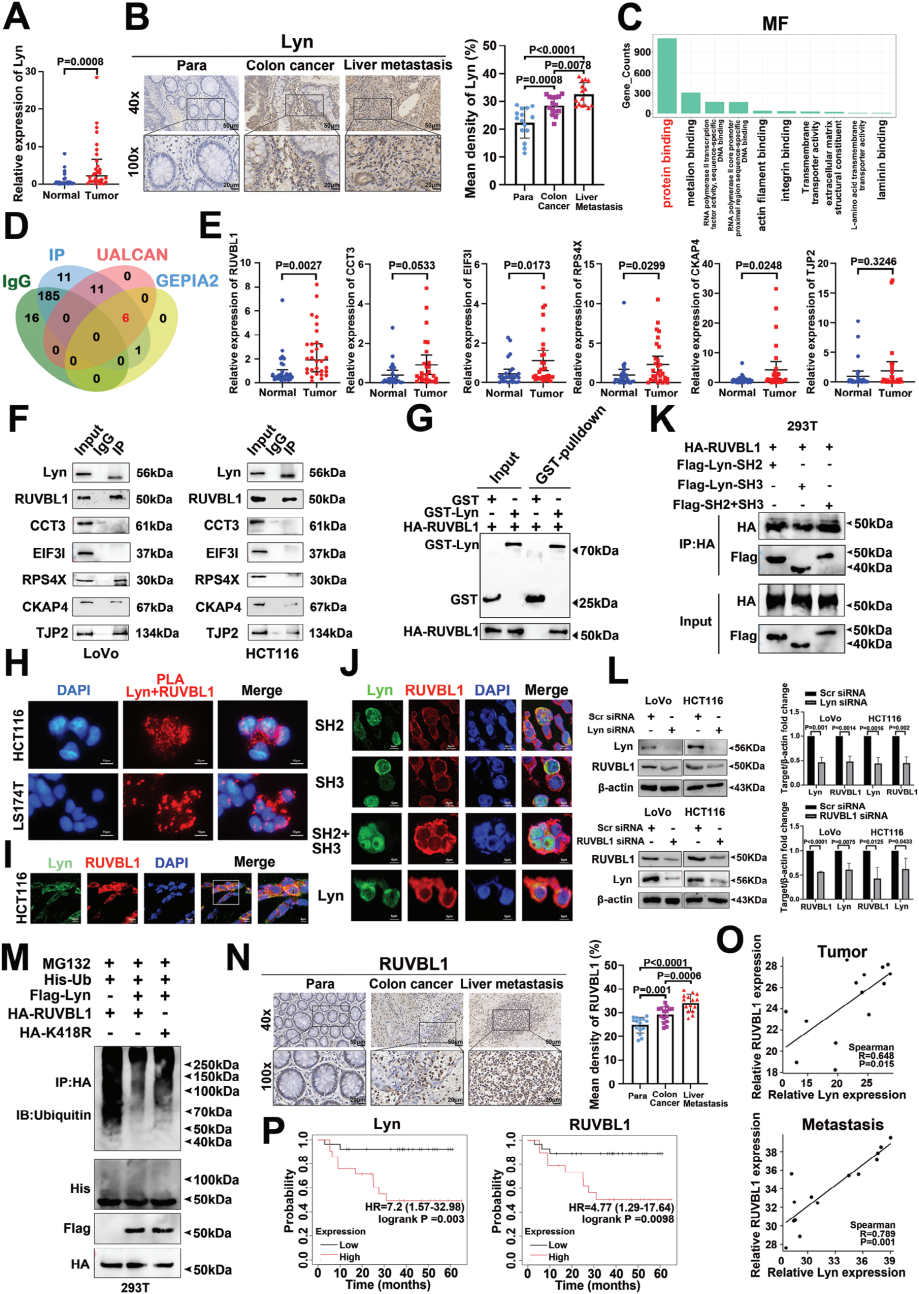
The Lyn and RUVBL1‐forming complex is highly expressed in CRC and is closely associated with liver metastasis. A) qRT‒PCR was employed to detect the expression of Lyn mRNA in tissues obtained from patients with CRC (*n* = 30). B) IHC was used to determine the expression of Lyn in normal tissues, CRC, and liver metastases (*n* = 15). C) MF by RNA‐seq differential gene GO analysis revealed the enrichment of Lyn. D) Proteomics combined with bioinformatics analysis was used to identify proteins that interact with Lyn. E) qRT–PCR was performed to assess the mRNA expression levels of genes that bind to Lyn in patients with CRC (*n* = 30). F) The interaction of the IP antibody targeting Lyn with specific proteins in colon cancer cells was detected. G) GST pull‐down was used to investigate the direct interaction between Lyn and RUVBL1. H) PLA study of Lyn and RUVBL1 interactions in situ. I) IF demonstrated the localization of both Lyn and RUVBL1 in HCT116 cells. J) IF revealed the localization patterns of the truncated forms of Lyn and RUVBL1 in 293T cells. K) Co‐IP confirmed the physical interaction between the truncated forms of Lyn and RUVBL1. L) Western blot analysis revealed the interregulation of Lyn and RUVBL1 in colon cancer cells. M) Effect of Lyn on RUVBL1 ubiquitination. N) IHC was conducted to evaluate the expression pattern of RUVBL1 across normal tissues, CRC tissues, and liver metastases (*n* = 15). O) Correlation analysis was used to investigate the relationships between the expression levels of both Lyn and RUVBL1 in CRC tissue samples and liver metastases. P) Lyn and RUVBL1 survival analysis in patients with colon cancer. Data are presented as the means ± SDs. For (A,B,E,L,N), unpaired *t*‐tests were performed. For (P), the log‐rank test was used.

Lyn is extensively utilized as a therapeutic target in various types of tumors.^[^ ^26^, ^27^, ^28^ ^]^ We detected the expression level of Lyn in CRC tissues and found that in cancer tissues, it was greater than that in normal adjacent tissues (**Figure** **1**A). Furthermore, immunohistochemical (IHC) analysis revealed that the expression level of Lyn in CRC tissues was significantly greater than that in adjacent normal tissues. In addition, its expression level in liver metastases was significantly greater than in normal tissues and primary CRC (Figure 1B). The TCGA database revealed that Lyn was highly expressed in colon cancer tissues compared with normal tissues at different stages (Figure S1A, Supporting Information). These results indicate that Lyn is highly expressed in CRC and is closely related to liver metastasis.

After Lyn was silenced in colon cancer cells, we performed RNA‐seq analysis. By conducting Gene Ontology (GO) on the downregulated differential genes identified via RNA‐seq, we found that the molecular function (MF) of these differential genes was enriched mainly in protein binding (Figure 1C). Subsequent NanoLC‐MS/MS analysis identified 236 proteins that interact with Lyn, with 29 specifically detected by Ip but not by IgG (Supplementary Table S1, Supporting Information). We combined bioinformatics analysis with GEPIA 2.0 and UALCAN database screening to identify six highly expressed proteins in colon cancer (Figure 1D; Figure S1B, Supporting Information). We detected the expression levels of these genes in tissues from CRC patients and found that they were highly expressed in cancer tissues, which was consistent with the database results (Figure 1E). The mass spectrometry results were confirmed with a silver stain, which revealed clear bands for the experimental and control groups that matched the Coomassie blue results (Figure S1C, Supporting Information). We used Lyn antibodies for Co‐IP and found that ruvB, like AAA ATPase 1 (RUVBL1), X‐linked ribosomal protein S4 (RPS4X), cytoskeleton‐associated protein 4 (CKAP4), and tight junction protein 2 (TJP2), could bind to Lyn, and RUVBL1 was most closely bound to Lyn (Figure 1F). Reverse validation via RUVBL1 antibodies confirmed that Lyn binds to RUVBL1 (Figure S1D, Supporting Information). GST pull‐down revealed that Lyn interacts directly with RUVBL1 (Figure 1G). The PLA experiment revealed an in situ interaction between Lyn and RUVBL1 (Figure 1H). Therefore, RUVBL1 was selected as the protein recruited by Lyn for further investigation.

Immunofluorescence (IF) demonstrated the colocalization of Lyn and RUVBL1 in colon cancer cell lines, CRC tissues, and CRC liver metastases (Figure 1I; Figure S1E,F, Supporting Information). Previous studies have indicated that the SH2 and SH3 domains of Lyn primarily facilitate PPIs.^[^ ^29^ ^]^ Consequently, we generated truncated forms of Lyn containing SH2, SH3, and SH2+SH3 domains with a Flag tag and a GFP tag, along with an overexpression plasmid for RUVBL1 tagged with an HA tag and an RFP tag (Figure S1G, Supporting Information). After cotransfection of the plasmids into 293T cells, RUVBL1 and Lyn SH2 and SH3 truncated bodies were expressed in both the nucleus and the cytoplasm (Figure 1J). Co‐IP confirmed that the Lyn SH2 and SH3 domains are combined with RUVBL1 (Figure 1K). The western blot results revealed that Lyn down‐regulated the expression of RUVBL1. Conversely, suppressing RUVBL1 in colon cancer cells also decreased Lyn levels (Figure 1L). The expression of RUVBL1 was upregulated after the overexpression of Lyn (Figure S1H, Supporting Information). Studies have shown that Lyn binds to IRF5 and that Lyn inhibits IRF5 ubiquitination and affects IRF5 expression.^[^ ^30^ ^]^ GART binds to RUVBL1, and the inhibition of RUVBL1 ubiquitination by GART affects the expression of RUVBL1.^[^ ^31^ ^]^ To further investigate the ability of Lyn to regulate RUVBL1, we searched the phosphosite database for ubiquitination at the K418 site bound to RUVBL1 by Lyn (Figure S1I, Supporting Information). The overexpression of Lyn inhibited RUVBL1 ubiquitination. After mutation of the K418 site, the ability of Lyn to inhibit RUVBL1 ubiquitination was weakened (Figure 1M). These results suggest that Lyn regulates RUVBL1 ubiquitination via K418. The use of GEPIA for the analysis of Lyn and RUVBL1 expression in the TCGA cohort revealed a positive correlation between their expression in CRC (Figure S1J, Supporting Information). Subsequent analysis of Lyn and RUVBL1 expression in tissues from CRC patients revealed a consistent positive correlation, which aligns with findings from the database (Figure S1K, Supporting Information). The level of RUVBL1 expression in CRC tissues was significantly greater than that in adjacent normal tissues. Furthermore, RUVBL1 expression was markedly increased in liver metastases (Figure 1N). Analysis of Lyn and RUVBL1 expression in CRC tissues and liver metastases revealed a positive correlation between these two proteins (Figure 1O). Survival analysis of stage 3 colon cancer patients via a Kaplan–Meier plotter revealed poor outcomes in patients with high expression of Lyn and RUVBL1 (Figure 1P). These results suggest that complexes formed by Lyn and RUVBL1 are highly expressed in CRC and closely associated with liver metastasis.

### 2.2 The Lyn/RUVBL1 Complex Promotes Invasion and Liver Metastasis of CRC

To investigate the impact of the Lyn/RUVBL1 complex on CRC function, we successfully engineered a recombinant plasmid based on the px330a dCas9‐KRAB plasmid as a vector for inhibiting Lyn expression (Figure S2A,B, Supporting Information). The lentivirus system was utilized to achieve stable inhibition of Lyn and RUVBL1 (Figure S2C–E, Supporting Information). CRISPR/Cas9 was employed to stably knock out Lyn and RUVBL1, and sgRNA2 was selected for subsequent studies (Figure S2F–I, Supporting Information).

**Figure 2:**
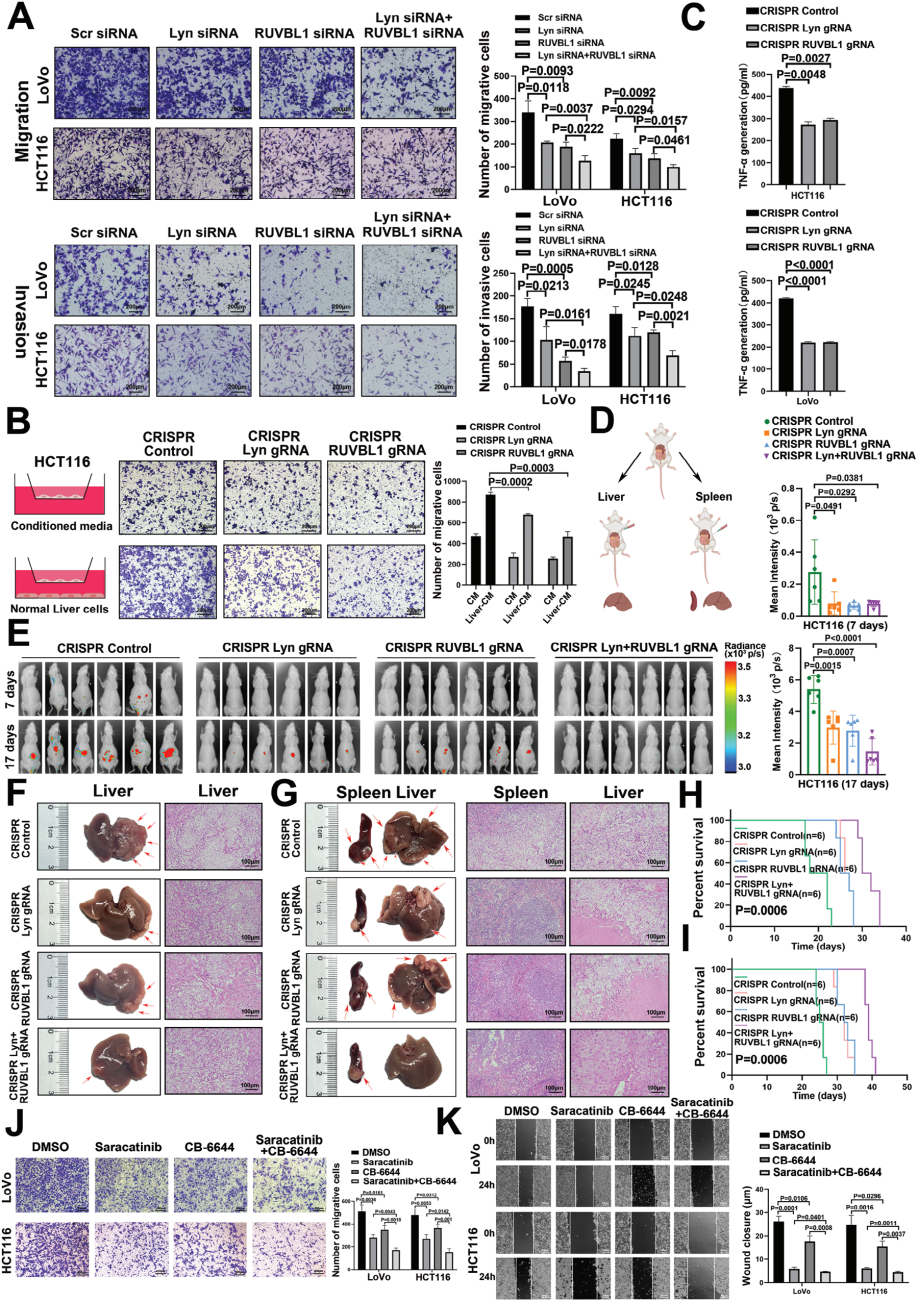
The Lyn/RUVBL1 complex promotes invasion and liver metastasis of CRC. A) The effect of the Lyn/RUVBL1 complex on the migration and invasion of colon cancer cells was detected via transwell assays. B) The effect of the Lyn/RUVBL1 complex on the hepatogenic migration of colon cancer cells was examined via coculture of colon cancer cells and hepatocytes in a Boyden chamber. C) Changes in TNF‐α levels in the supernatants of colon cancer cells with Lyn/RUVBL1 complex deletion cocultured with THLE‐2 cells. D) Diagram of the liver metastasis model of CRC in nude mice. E) Bioluminescence imaging was used to detect fluorescence intensity and quantify fluorescence in a liver metastasis model of CRC in nude mice (*n* = 6). F) Tumor formation and HE staining of the livers of nude mice inoculated with CRISPR/Cas9 to knock out Lyn and RUVBL1 in HCT116 cells. G) Tumor formation and HE staining of the spleen and liver of a nude mouse spleen inoculated with CRISPR/Cas9 to knock out the HCT116 cells of Lyn and RUVBL1. H) Survival analysis of HCT116 cells in which Lyn and RUVBL1 were knocked out via CRISPR/Cas9 in the livers of nude mice. I) Survival analysis of a nude mouse spleen inoculated with CRISPR/Cas9‐knockout HCT116 cells harboring Lyn and RUVBL1. J) Transwell analysis of the effects of the drug intervention Lyn/RUVBL1 on colon cancer cell function. K) The effect of the drug intervention Lyn/RUVBL1 on colon cancer cell function was analyzed via a scratch assay. Data are presented as the means ± SDs. Unpaired *t*‐tests were performed for (A–C,E,J,K). For (H,I), the log‐rank test was used.

A Cell Counting Kit‐8 (CCK‐8) assay revealed that the proliferation ability of colon cancer cells was significantly reduced after Lyn knockout (Figure S3A, Supporting Information). Scratch assays revealed that colon cancer cell migration was inhibited after Lyn knockout (Figure S3B, Supporting Information). We found that inhibiting Lyn and RUVBL1 significantly reduced colon cancer cell migration and invasion. Compared with individual inhibition, simultaneous inhibition of both genes had a significant effect (**Figure** **2**A; Figure S3C, Supporting Information). Through a rescue experiment, we found that the overexpression of Lyn enhanced the migration and invasion of colon cancer cells, whereas the inhibition of RUVBL1 reversed this effect (Figure S3D,E, Supporting Information). To determine whether Lyn/RUVBL1 mediates the metastasis of CRC to the liver, we used the Boyden coculture system and found that the conditioned medium from normal liver cells notably facilitated CRC metastasis. This hepatic tropism was attenuated in Lyn/RUVBL1‐deficient cells (Figure 2B). Furthermore, analysis of TNF‐α chemokine levels in the medium revealed a decrease in supernatant TNF‐α levels following the inhibition of Lyn and RUVBL1 (Figure 2C).

Stable colon cancer cell lines were generated and injected into nude mice to study the effect of the Lyn/RUVBL1 complex on CRC metastasis (Figure 2D). Bioluminescence imaging revealed a significant reduction in the fluorescence signal in nude mice following liver inoculation with colon cancer cells in which Lyn and RUVBL1 were silenced (Figure 2E; Figure S3F, Supporting Information). The control group of liver‐inoculated nude mice presented a high number of visible tumor nodules in the liver, whereas the nude mice with stable silencing of Lyn and RUVBL1 colon cancer cells presented a lower number of tumor nodules in the liver (Figure 2F; Figure S3G, Supporting Information). Tumor nodules were abundant in both the spleen and liver of nude mice inoculated with the spleen. Nude mice inoculated with stably silenced Lyn and RUVBL1 colon cancer cell lines developed tumor nodules in the spleen, along with a few visible tumor nodules in the liver (Figure 2G; Figure S3H–K, Supporting Information). Survival analysis revealed that compared with control nude mice, nude mice inoculated with silenced Lyn and RUVBL1 colon cancer cells had significantly longer lifespans (Figure 2H,I; Figure S3L,M, Supporting Information). Saracatinib is an inhibitor of Lyn,^[^ ^32^ ^]^ and CB‐6644 is an inhibitor of RUVBL1.^[^ ^33^ ^]^ The results revealed that the migration and invasion ability of colon cancer cells was significantly reduced after drug intervention in the Lyn/RUVBL1 complex (Figure 2J–K). These results indicate that the Lyn/RUVBL1 complex promotes invasion and liver metastasis in CRC.

### 2.3 The Lyn/RUVBL1 Complex Enhances the Liver Metastasis of CRC by Modulating the Chromatin Accessibility of β‐Catenin

**Figure 3:**
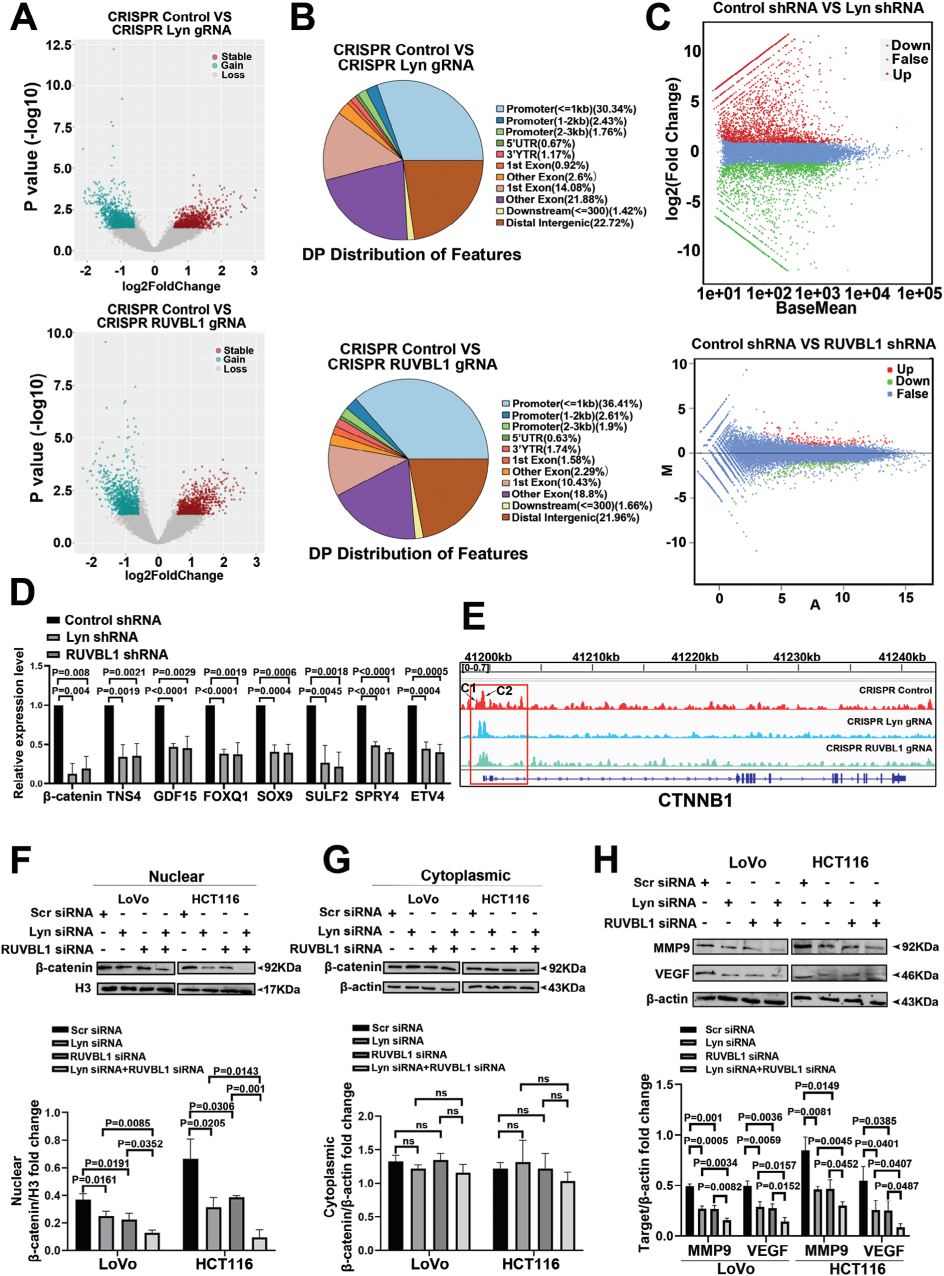
The Lyn/RUVBL1 complex enhances the liver metastasis of CRC by modulating the chromatin accessibility of β‐catenin. A) ATAC‐seq was used to detect differential genes obtained after CRISPR/Cas9 knockout of the Lyn/RUVBL1 complex. B) ATAC‐seq was used to detect the distribution of peak differences in the genome after CRISPR/Cas9 knockout of Lyn and RUVBL1. C) RNA‐seq detection of differentially expressed genes obtained after lentiviral knockdown of the Lyn/RUVBL1 complex. D) qRT‒PCR validated Lyn and RUVBL1 RNA‐seq downregulation and genes associated with CRC. E) Changes in the chromatin accessibility of β‐catenin after Lyn/RUVBL1 knockout were detected via ATAC‐seq. F) The expression of β‐catenin in the nucleus and the statistical results of grayscale analysis after Lyn and RUVBL1 knockdown were detected using Western blotting. G) Western blotting was used to detect the expression of β‐catenin in the cytoplasm after Lyn and RUVBL1 knockdown, and the statistical results of grayscale analysis are shown. H) Western blotting was used to detect the expressions of MMP9 and VEGF after Lyn and RUVBL1 were knocked down, and the statistical results of grayscale analysis were obtained. Data are presented as the means ± SDs. For (D) and (F–H), an unpaired *t*‐test was used.

We employed ATAC‐seq to investigate the involvement of the Lyn/RUVBL1 complex in chromatin remodeling. Chromatin accessibility was increased in 928 genes and decreased in 1147 genes following Lyn knockout. After RUVBL1 knockout, chromatin accessibility was increased in 933 genes and decreased in 1195 genes (**Figure** **3**A). The majority of the observed changes were located in the promoter and distal intergenic regions (Figure 3B). Lyn RNA‐seq revealed 2578 upregulated genes and 2020 downregulated genes. RUVBL1 RNA‐seq revealed 156 upregulated genes and 151 downregulated genes (Figure 3C). qRT‒PCR was used to verify genes codownregulated in the RNA‐seq results of Lyn and RUVBL1 and genes associated with CRC, and β‐catenin was found to be the most significantly downregulated gene (Figure 3D). Visual analysis revealed a decrease in the chromatin accessibility of β‐catenin in colon cancer cells following the suppression of Lyn/RUVBL1 (Figure 3E).

Multiple studies have shown that MMP9 and VEGF promote liver metastasis in CRC.^[^ ^34^, ^35^, ^36^ ^]^ Our previous research demonstrated that DC‐SIGN induces the nuclear translocation of β‐catenin by recruiting Lyn to form protein complexes, thereby increasing the expression of MMP9 and VEGF and promoting the migration and invasion of colon cancer cells.^[^ ^37^ ^]^ Compared with the control, Lyn, RUVBL1, or both considerably inhibited the nuclear translocation of β‐catenin (Figure 3F,G). Additionally, the expression levels of MMP9 and VEGF decreased after Lyn/RUVBL1 knockdown (Figure 3H). These findings suggest that Lyn/RUVBL1 may play a role in promoting CRC liver metastasis by influencing β‐catenin chromatin accessibility.

### 2.4 The Lyn/RUVBL1 Complex Mediates Chromatin Remodeling Through RNA Polymerase II and Activates TRIB3 to Drive β‐Catenin to Promote CRC Metastasis

We designed primers in the promoter region of β‐catenin. The results of ChIP‒qPCR revealed that Lyn/RUVBL1 did not bind to the promoter region of β‐catenin (Figure S4A, Supporting Information). To further investigate the mechanism of action of Lyn/RUVBL1 on β‐catenin, after Lyn/RUVBL1 knockdown, we screened for DNA interactions mediated by chromatin remodeling‐associated proteins. The results revealed a decrease in the expression of chromatin remodeling‐related proteins, with the largest catalytic subunit of RNA polymerase II (POLR2A) showing the most significant decrease (Figure S4B, Supporting Information). GO analysis of downregulated genes from Lyn ATAC‐seq and RNA‐seq in LoVo revealed that the biological process (BP) was related mainly to the regulation of RNA polymerase II promoter transcription (Figure S4C, Supporting Information). GO analysis of downregulated genes from Lyn and RUVBL1 RNA‐seq revealed that the cellular component (CC) was predominantly linked to the nucleus and chromatin, with BP associated primarily with the regulation of RNA polymerase II promoter transcription (Figure S4D, Supporting Information).

**Figure 4:**
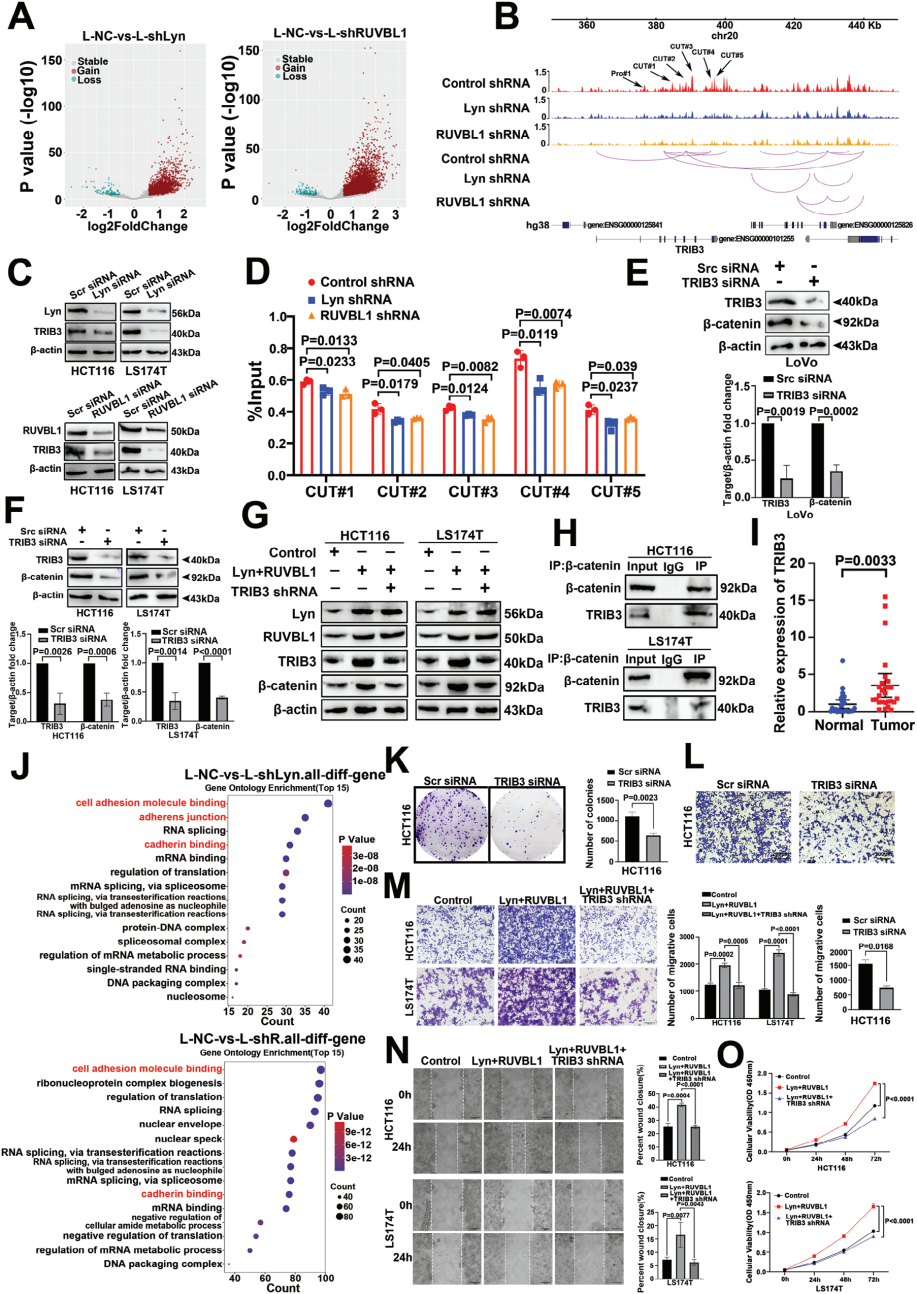
The Lyn/RUVBL1 complex mediates chromatin remodeling through RNA polymerase II and activates TRIB3 to drive β‐catenin to promote CRC metastasis. A) HiCuT was used to detect differential genes obtained after lentiviral knockdown of the Lyn/RUVBL1 complex. B) Visual analysis of the TRIB3 gene with a reduced differential peak on the differential loop after the knockdown of Lyn and RUVBL1. C) Western blot analysis of the effect of the Lyn/RUVBL1 complex on TRIB3 protein levels in colon cancer cell lines. D) The effect of the Lyn/RUVBL1 complex on the binding ability of TRIB3 and RNA polymerase II interaction sites was detected by ChIP‒qPCR. E) Western blot analysis of the effects of TRIB3 on β‐catenin protein levels in LoVo cells. F) Western blot analysis of the effects of TRIB3 on β‐catenin protein levels in HCT116 and LS174T cells. G) Western blot analysis of β‐catenin expression after TRIB3 was knocked down and Lyn/RUVBL1 overexpression. H) The interaction between TRIB3 and β‐catenin was studied via IP. I) qRT‒PCR was used to detect the expression of TRIB3 mRNA in the tissues of CRC patients. J) HiCuT was used for GO analysis of differentially expressed genes. K) The effects of TRIB3 on the proliferation of HCT116 cells were detected via plate cloning. L) The effect of TRIB3 on the migration and invasion of HCT116 cells was detected via transwell assays. M) Transwell assays were used to examine the effect of TRIB3 knockdown on the invasion of colon cancer cells after Lyn/RUVBL1 overexpression. N) The effect of TRIB3 knockdown on colon cancer cells migration was examined in the context of Lyn/RUVBL1 overexpression using a scratch assay. O) The effect of TRIB3 knockdown on colon cancer cells proliferation was examined in the context of Lyn/RUVBL1 overexpression via a CCK8 assay. Data are presented as the means ± SDs. For (D,E–F,I,K–N), unpaired *t*‐tests were performed. For (O), two‐way ANOVA was used.

RUVBL1 has been reported to be able to directly control Pol II transcription factories near active promoters.^[^ ^38^ ^]^ HiCuT experiments investigated the involvement of Lyn/RUVBL1 in Pol II‐mediated chromatin loops. DESeq2 was used to analyze the differential peak (DP) of HiCuT (**Figure** **4**A), whereas the diffloop R package was used for the differential analysis of the HiCuT data (Figure S4E, Supporting Information). Visualization analysis was performed on genes showing reduced DPs and differential loops after Lyn/RUVBL1 knockdown, revealing a significant decrease in TRIB3 (Figure 4B). Western blot analysis revealed TRIB3 downregulation after Lyn/RUVBL1 knockdown (Figure 4C; Figure S4F, Supporting Information). The primers used were designed at the downregulated position of the TRIB3 peak and ChIP–qPCR demonstrated a weakened interaction between Pol II and TRIB3 following the knockdown of Lyn/RUVBL1 (Figure 4D). ChIP‒qPCR revealed that Lyn/RUVBL1 did not directly bind to the promoter region of TRIB3 (Figure S4G, Supporting Information).

Western blot analysis revealed that β‐catenin was downregulated after TRIB3 knockdown (Figure 4E,F). β‐catenin expression was increased when Lyn/RUVBL1 was overexpressed and decreased when Lyn/RUVBL1 was overexpressed and TRIB3 was knocked down (Figure 4G; Figure S4H, Supporting Information). These results suggest that Lyn/RUVBL1 regulates β‐catenin expression through TRIB3. Research has shown that TRIB3 physically interacts with β‐catenin and activates it in various types of tumors.^[^ ^39^, ^40^, ^41^ ^]^ Co‐IP revealed that TRIB3 interacts with β‐catenin in colon cancer cells (Figure 4H). TCGA indicated elevated expression of TRIB3 in colon cancer tissues (Figure S4I, Supporting Information). qRT–PCR was utilized to assess the expression level of TRIB3 in tissues from CRC patients, revealing higher expression levels in cancerous tissues than in adjacent normal tissues (Figure 4I). GO analysis revealed that the knockdown of Lyn/RUVBL1 resulted in significant enrichment in cell adhesion, a process closely associated with tumor metastasis (Figure 4J). Plate clone and transwell assays demonstrated that TRIB3 promoted the proliferation, invasion, and metastasis of colon cancer cells (Figure 4K,L). Lyn/RUVBL1 regulates β‐catenin via TRIB3. The results of functional experiments revealed that the proliferation, migration, and invasion functions of colon cancer cells were enhanced after the overexpression of Lyn/RUVBL1, and the proliferation, migration, and invasion functions of colon cancer cells were weakened after the overexpression of Lyn/RUVBL1 knocked down TRIB3 (Figure 4M–O). These findings suggest that Lyn/RUVBL1 mediates chromatin remodeling through RNA polymerase II and activates TRIB3, ultimately driving β‐catenin to promote CRC metastasis.

### 2.5 The Lyn/RUVBL1 Complex Increases the Metabolic Reprogramming of AA to Promote CRC Liver Metastasis

**Figure 5:**
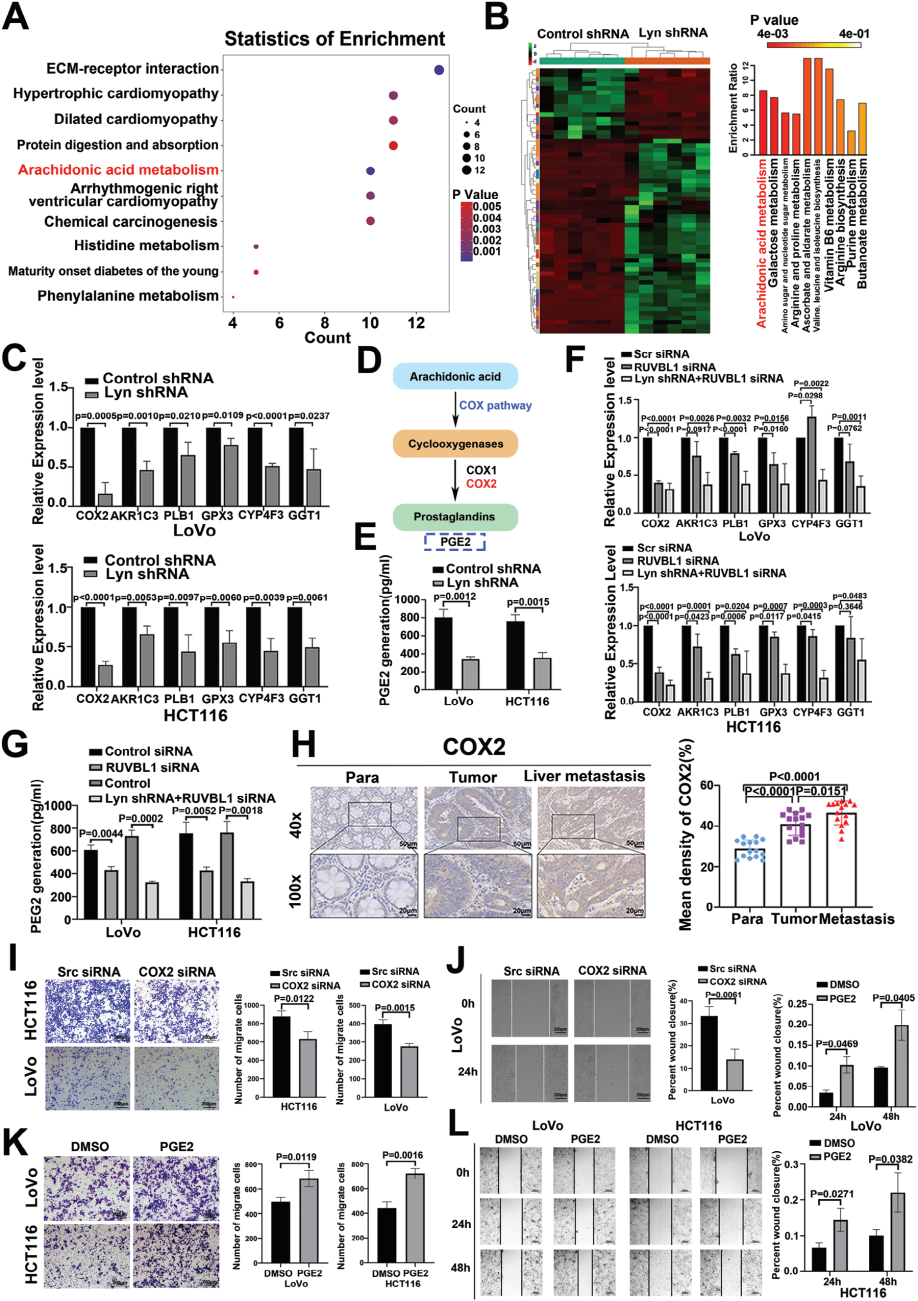
The Lyn/RUVBL1 complex increases the metabolic reprogramming of AA to promote CRC liver metastasis. A) KEGG enrichment analysis of genes downregulated in Lyn according to RNA‐seq. B) Cluster heatmap of metabolomic differentially abundant metabolites (left). KEGG enrichment analysis of downregulated differentially abundant metabolites (right). C) qRT‒PCR was used to detect the mRNA expression levels of key genes involved in arachidonic acid metabolism in colon cancer cells after Lyn knockdown. D) Schematic of the AA metabolic pathway. E) The expression level of PGE2 after the knockdown of Lyn was detected via ELISA. F) qRT‒PCR was used to detect the mRNA expression levels of key genes of the arachidonic acid metabolic pathway in colon cancer cells after RUVBL1, Lyn, and RUVBL1 knockdown. G) ELISA was used to detect PGE2 expression levels after RUVBL1 was knocked down and Lyn and RUVBL1 were knocked down. H) The expression of COX2 in adjacent normal tissues, CRC tissues, and liver metastases was detected by IHC (*n* = 15). I) Transwell assays were used to detect the effects of COX2 on the migration and invasion of colon cancer cells. J) The effect of COX2 on the migration ability of LoVo cells was detected via a scratch test. K) The effect of PGE2 on colon cancer cell invasion was detected using a transwell assay. L) The effect of PGE2 on colon cancer cell migration was detected using a scratch assay. Data are presented as the means ± SDs. For (C,E–L), an unpaired *t*‐test was used.

Kyoto Encyclopedia of Genes and Genomes (KEGG) enrichment analysis of ATAC‐seq data following the inhibition of Lyn expression revealed that differentially expressed genes were predominantly enriched in metabolic pathways (Figure S5A, Supporting Information). Lyn ATAC‐seq and RNA‐seq revealed that differentially expressed genes were also enriched mainly in metabolic pathways (Figure S5B, Supporting Information). KEGG enrichment analysis of Lyn RNA‐seq data demonstrated that the differentially expressed genes were enriched primarily in AA metabolism (**Figure** **5**A). Widely targeted metabolomics revealed that 55 metabolites were downregulated and that 177 metabolites were upregulated after Lyn knockdown. KEGG enrichment analysis of the downregulated metabolites indicated that the differentially expressed metabolites were enriched primarily in the AA metabolic pathway (Figure 5B). According to the Lyn RNA‐seq results, molecules related to AA metabolism were selected for verification, revealing a considerable decrease in the expression of cyclooxygenase‐2 (COX2), a key enzyme in AA metabolism (Figure 5C). Prostaglandin E2 (PGE2) is the main metabolite of AA catalyzed by COX2, and its expression is decreased according to metabolomics (Figure 5D; Figure S5C, Supporting Information). ELISA revealed a significant decrease in PGE2 after Lyn knockdown (Figure 5E), whereas PGE2 expression increased after Lyn overexpression (Figure S5D, Supporting Information).

Combined analysis of the RNA‐seq data of Lyn and RUVBL1 revealed that the downregulated differentially expressed genes were related mainly to metabolic pathways (Figure S5E, Supporting Information). After RUVBL1 was knocked down and Lyn and RUVBL1 were knocked down, the expressions of COX2 and PGE2 decreased (Figure 5F,G). IHC revealed that the expression of COX2 in CRC tissues was greater than that in adjacent normal tissues. COX2 expression was significantly greater in liver metastases than in normal tissues and primary CRC tissues (Figure 5H). Scratch and transwell assays revealed that COX2 promoted the invasion and metastasis of colon cancer cells (Figure 5I,J). COX2 upregulates PGE2. PGE2 was added to the cells at the reported concentration.^[^ ^42^ ^]^ Scratch and transwell assays revealed that PGE2 promoted the invasion and metastasis of colon cancer cells (Figure 5K,L). These findings suggest that the Lyn/RUVBL1 complex regulates the metabolic reprogramming of AA and promotes the invasion and liver metastasis of CRC.

### 2.6 The Lyn/RUVBL1 Complex Regulates AA Metabolism in Colon Cancer Cells Via the Upregulation of COX2 Through FOXA1

**Figure 6:**
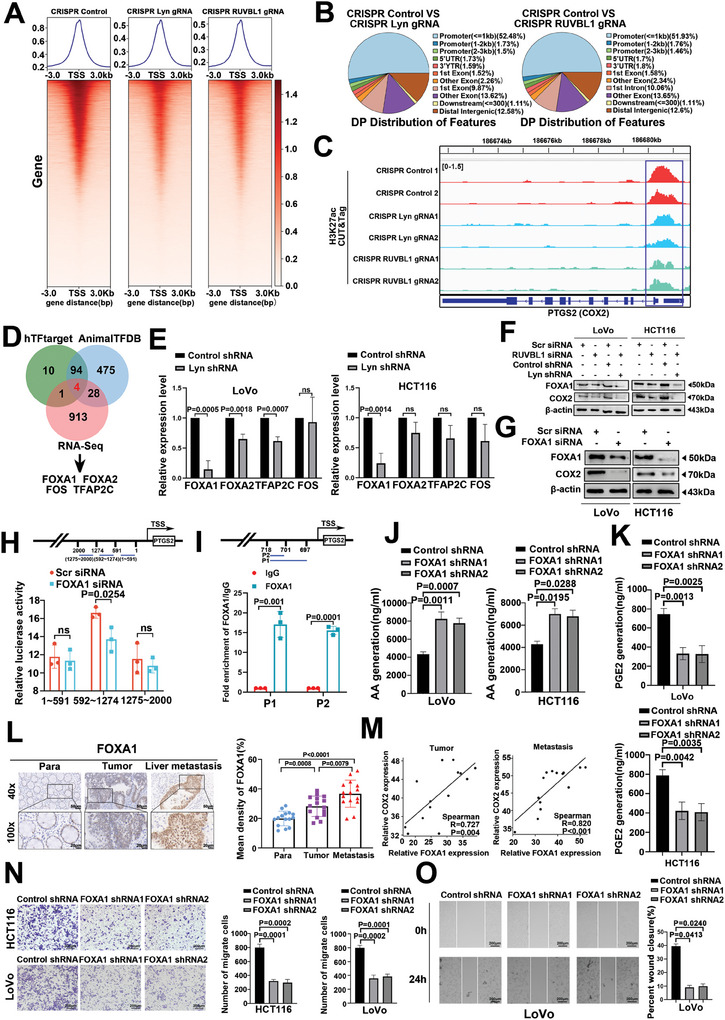
The Lyn/RUVBL1 complex regulates AA metabolism in colon cancer cells via the upregulation of COX2 through FOXA1. A) CUT‐Tag was used to detect TSS enrichment of H3K27ac after CRISPR/Cas9 knockout of Lyn and RUVBL1. B) CUT‐Tag was used to detect the distribution of peak differences in the genome after CRISPR/Cas9 knockout of Lyn and RUVBL1. C) Kurtosis diagram of the CUT‐Tag of PTGS2. D) Venn diagram of hTFtarget, AnimalTFDB, and Lyn RNA‐seq data. E) qRT‒PCR was used to detect the mRNA expression of related molecules after Lyn was knocked down in colon cancer cells. F) Western blot analysis of the effects of the Lyn/RUVBL1 complex on COX2 and FOXA1 protein levels in colon cancer cell lines. G) Western blot analysis of the protein expression level of COX2 in colon cancer cells after FOXA1 knockdown. H) A dual‐luciferase reporter gene assay was used to detect luciferase activity in different regions of the COX2 promoter after FOXA1 was knocked down. I) The binding sites of FOXA1 and COX2 were detected by ChIP‒qPCR. J) The expression level of AA after FOXA1 knockdown was detected using ELISA. K) The expression level of PGE2 after FOXA1 knockdown was detected via ELISA. L) FOXA1 expression in adjacent normal, CRC, and liver metastasis tissues was detected via IHC (*n* = 15). M) Correlation analysis of FOXA1 and COX2 expression in CRC and liver metastases. N) Transwell assays were used to detect the effects of FOXA1 on the migration and invasion of colon cancer cells. O) The effect of FOXA1 on the migration ability of LoVo cells was detected using a scratch assay. Data are presented as the means ± SDs. For (E,H–L,N,O), unpaired *t*‐tests were performed.

RUVBL1 is a component of INO80‐C. INO80‐C and the H3K27 acetyltransferase P300 physically interact, suggesting that INO80‐C and P300 may jointly coordinate chromatin accessibility at canonical INO80 sites.^[^ ^43^ ^]^ After Lyn and RUVBL1 were knocked out, the expressions of P300 and H3K27ac decreased (Figure S6A, Supporting Information). P300 downregulates the expression of H3K27ac (Figure S6B, Supporting Information). After the overexpression of Lyn/RUVBL1, the expression of H3K27ac increased, and after the overexpression of Lyn/RUVBL1 knocked down P300, the expression of H3K27ac decreased (Figure S6C, Supporting Information). These results show that Lyn/RUVBL1 regulates H3K27ac through P300. To further investigate the effects of Lyn/RUVBL1 on the epigenetics of colon cancer cells, we examined whole‐genome H3K27ac levels using CUT‐Tag. TSS enrichment was weakened after the inhibition of Lyn and RUVBL1 (**Figure** **6**A). The observed peak difference was predominantly localized to the promoter (Figure 6B). IGV revealed that H3K27ac levels in the COX2 promoter were downregulated after Lyn and RUVBL1 were knocked down (Figure 6C). We utilized hTFtarget, AnimalTFDB, and Lyn RNA‐seq to identify COX2 transcription factors, resulting in the identification of FOXA1, FOXA2, FOS, and TFAP2C (Figure 6D). The hTFtarget score was combined with motif analysis of Lyn ATAC‐seq and HiCut to assess the enrichment level of transcription factor motifs in relevant genomic regions. Notably, FOXA1 presented the highest score and lowest *p*‐value (Figure S6D, Supporting Information). qRT‒PCR revealed that FOXA1 was most considerably decreased in colon cancer cells after Lyn knockdown (Figure 6E). Additionally, our findings demonstrated that Lyn/RUVBL1 enhances the expressions of COX2 and FOXA1 (Figure 6F; Figure S6E, Supporting Information). COX2 mRNA and protein levels were significantly decreased after FOXA1 knockdown (Figure 6G; Figure S6F,G, Supporting Information). When Lyn was overexpressed in colon cancer cells, Lyn facilitated COX2, whereas the inhibition of FOXA1 reversed this effect (Figure S6H, Supporting Information). The plasmid was constructed for a dual‐luciferase reporter gene assay in which the first 2000 bp of the COX2 promoter region was used to assess changes in luciferase activity. The results revealed that knocking down FOXA1 in the COX2 promoter region between 592 and 1274 bp could reduce luciferase activity (Figure 6H). The binding sites of FOXA1 and COX2 were predicted by the hTFtarget database to be between 699 and 718 bp (Figure S6I, Supporting Information). We then designed the primers. The ChIP‒qPCR results revealed that FOXA1 was bound to the promoter of COX2 (Figure 6I). The results showed that FOXA1 is a transcription factor of COX2.

AA conversion to PGE2 is catalyzed by COX2. ELISA revealed that after FOXA1 inhibition, AA expression was upregulated (Figure 6J), and PGE2 expression was downregulated (Figure 6K). These results suggested that the metabolic pathway of arachidonic acid was weakened after FOXA1 inhibition. IHC revealed that the level of FOXA1 expression in CRC tissues was greater than that in normal tissues. FOXA1 was significantly elevated in liver metastases compared with normal tissue and primary CRC (Figure 6L). Analysis of the expressions of COX2 and FOXA1 in CRC tissues and liver metastases revealed a positive correlation between the two proteins (Figure 6M). The effect of FOXA1 on the function of colon cancer cells was examined through lentivirus‐mediated inhibition of FOXA1 expression (Figure S6J, Supporting Information). Transwell and scratch assays demonstrated that FOXA1 promoted the invasion and metastasis of colon cancer cells (Figure 6N,O; Figure S6K, Supporting Information). In summary, the Lyn/RUVBL1 complex regulates AA metabolism in colon cancer cells by upregulating COX2 through FOXA1.

### 2.7 The Lyn/RUVBL1 Complex Facilitates the Nuclear Translocation of β‐Catenin to Upregulate MMP9 and VEGF by Regulating AA Metabolism in CRC

**Figure 7:**
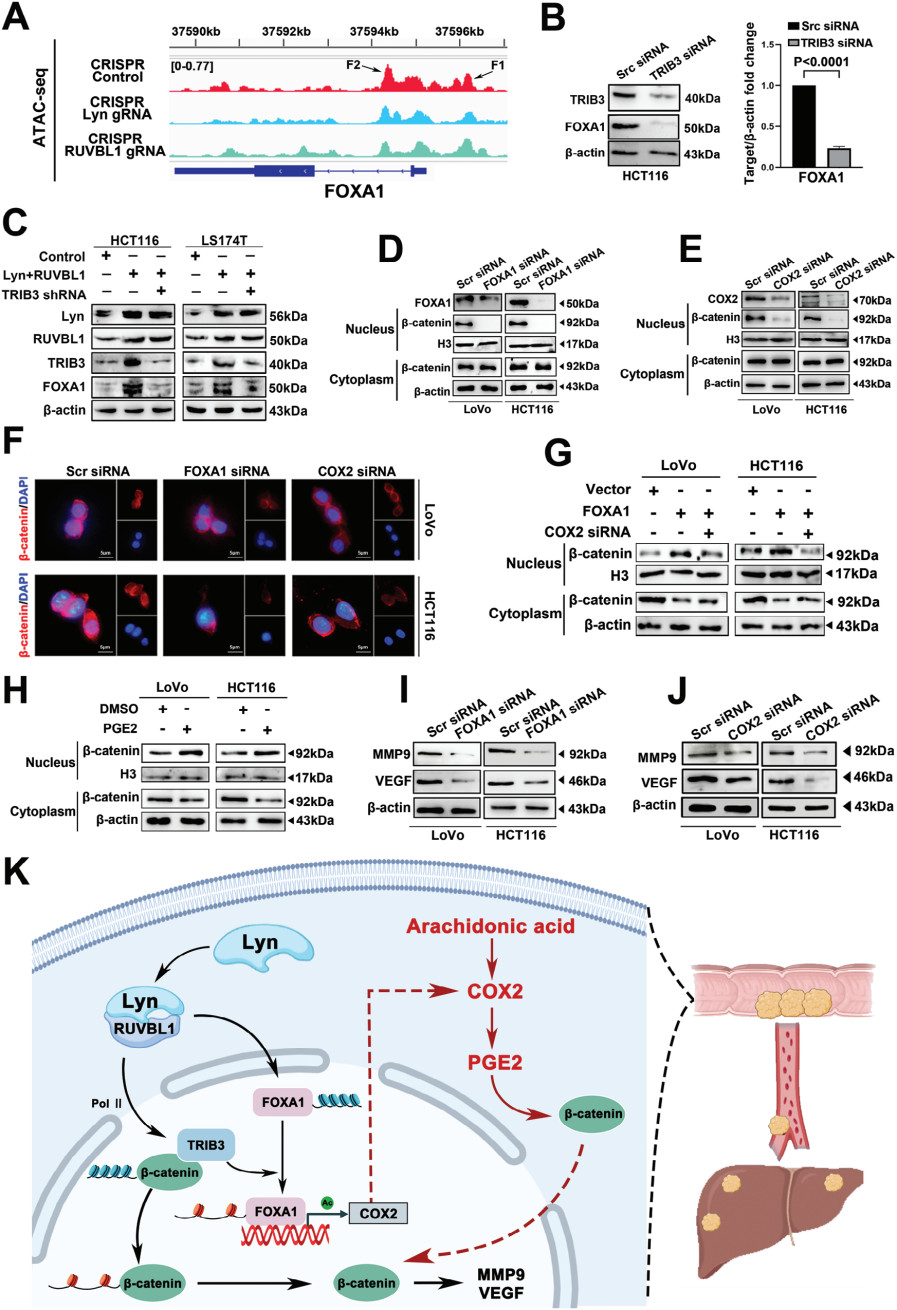
The Lyn/RUVBL1 complex facilitates the nuclear translocation of β‐catenin to upregulate MMP9 and VEGF by regulating AA metabolism in CRC. A) Changes in FOXA1 chromatin accessibility after Lyn/RUVBL1 knockout were detected via ATAC‐seq. B) Western blot analysis of the effect of TRIB3 knockdown on the FOXA1 protein in HCT116 cells. C) Western blot analysis of FOXA1 expression after Lyn/RUVBL1 was overexpressed and TRIB3 was knocked down. D) Western blot analysis of β‐catenin expression in colon cancer cells after FOXA1 knockdown. E) Western blot analysis of β‐catenin expression after COX2 knockdown in colon cancer cells. F) The effects of FOXA1 and COX2 knockdown on the subcellular localization of β‐catenin were detected by IF. G) Western blot detection of β‐catenin expression after FOXA1 was overexpressed and COX2 was knocked down. H) The effect of PGE2 on β‐catenin was detected by Western blot. I) Western blot analysis of the protein expression levels of MMP9 and VEGF in colon cancer cells after FOXA1 knockdown. J) Western blot analysis of the expression levels of MMP9 and VEGF in colon cancer cells after COX2 knockdown. K) Lyn/RUVBL1 complex‐mediated chromatin remodeling regulates arachidonic acid metabolism and promotes CRC liver metastasis. Data are presented as the means ± SDs. For (B), an unpaired *t*‐test was used.

ATAC‐seq revealed a decrease in the chromatin accessibility of FOXA1 following Lyn/RUVBL1 knockout (**Figure** **7**A). We designed primers in areas where FOXA1 chromatin changes. ChIP‒qPCR revealed that Lyn/RUVBL1 did not directly bind FOXA1 (Figure S7A, Supporting Information). KEGG analysis revealed that the HiCuT differential genes of Lyn/RUVBL1 were enriched in fatty acid biosynthesis (Figure S7B, Supporting Information). Additionally, FOXA1 was downregulated after TRIB3 was knocked down in HCT116 cells (Figure 7B). Western blot analysis revealed that FOXA1 expression increased after the overexpression of Lyn/RUVBL1 and decreased after TRIB3 knockdown (Figure 7C; Figure S7C, Supporting Information). Lyn/RUVBL1 regulates FOXA1 through TRIB3. We discovered that Lyn/RUVBL1 activates TRIB3 to drive β‐catenin. Western blot analysis revealed that knocking down FOXA1 and COX2 significantly inhibited the nuclear translocation of β‐catenin (Figure 7D,E; Figure S7D,E, Supporting Information). IF revealed that in most cells in the experimental group, β‐catenin was localized in the cytoplasm, whereas in the control group, free β‐catenin accumulated in the cytoplasm and translocated to the nucleus (Figure 7F). To further investigate whether FOXA1 promotes β‐catenin entry into the nucleus through COX2, β‐catenin entry into the nucleus increased after FOXA1 was overexpressed, whereas β‐catenin entry decreased after overexpression of FOXA1 and knockdown of COX2 (Figure 7G; Figure S7F, Supporting Information). The results showed that FOXA1 promoted the entry of β‐catenin into the nucleus through COX2. Several studies have shown that PGE2 can promote the entry of β‐catenin into the nucleus.^[^ ^44^, ^45^, ^46^ ^]^ COX2 promotes the expression of PGE2. Western blot analysis revealed that β‐catenin increased in the nucleus of colon cancer cells after PGE2 addition (Figure 7H; Figure S7G, Supporting Information). The results showed that COX2 promoted the entry of β‐catenin into the nucleus through PGE2. Further research revealed that the levels of MMP9 and VEGF decreased after FOXA1 and COX2 were downregulated (Figure 7I,J; Figure S7H,I, Supporting Information). These findings indicate that the Lyn/RUVBL1 complex drives β‐catenin nuclear translocation to upregulate MMP9 and VEGF by modulating AA metabolism in colon cancer cells.

## 3 Discussion

Epigenetic remodeling and metabolic reprogramming are closely related and regulate each other, serving as well‐established markers of cancer.^[^ ^13^ ^]^ The transition from a healthy state to metastatic cancer is predominantly governed by epigenetic alterations.^[^ ^47^ ^]^ Metabolic reprogramming provides energy for tumor cell survival and proliferation. In this study, we linked epigenetic remodeling and metabolic reprogramming to describe the role of the Lyn/RUVBL1 complex in CRC invasion and liver metastasis.

Lyn reportedly forms protein complexes with EPHA2, regulating epithelial–mesenchymal plasticity and metastasis in breast cancer.^[^ ^24^ ^]^ Additionally, Lyn interacts with CD24 to activate ERK1/2 and promote CRC metastasis.^[^ ^25^ ^]^ Our study revealed the interaction between Lyn and RUVBL1 in CRC. RUVBL1 is a highly conserved AAA+ ATPase.^[^ ^48^ ^]^ LINC00839 in colon cancer cells recruits RUVBL1 into the Tip60 complex, leading to increased acetylase activity. This upregulates the expression of nuclear respiratory factor 1 and activates mitochondrial metabolism, thereby promoting the progression of CRC.^[^ ^49^ ^]^ We found increased expression of Lyn and RUVBL1 in the tissues of patients with CRC, especially those with CRC liver metastasis. Functional experiments conducted in vivo and in vitro demonstrated that inhibition of Lyn and RUVBL1 expression led to suppressed growth and liver metastasis of CRC. These findings support the hypothesis that Lyn/RUVBL1 may play a pivotal role in the liver metastasis of CRC.

Lyn is a member of the SFK family expressed in all blood cells except T lymphocytes and was the first SFK family found to play a dual role.^[^ ^50^ ^]^ The SH2 and SH3 domains of the SFK are closely associated with PPIs.^[^ ^51^ ^]^ EGF regulates the interaction between Src kinase and tyrosine kinase substrates by modulating the SH2 and SH3 domains of Src kinase.^[^ ^52^ ^]^ Studies have demonstrated that the inhibition of the Lyn SH2 and SH3 domains can attenuate the interaction between Lyn and EGFP.^[^ ^53^ ^]^ We generated Lyn SH2, SH3, and SH2+SH3 truncated plasmid and RUVBL1 overexpression plasmids to assess the specific binding of Lyn to RUVBL1. The results of IF and Co‐IP revealed that RUVBL1 combined with the SH2 and SH3 domains of Lyn, which was consistent with the above results.

Lyn mediates the separation of A‐Kinase Anchoring Protein 8 from chromatin and the nuclear matrix in a manner dependent on kinase activity, thereby modulating chromatin structural changes.^[^ ^54^ ^]^ RUVBL1 is a component of the INO80 chromatin remodeling complex,^[^ ^55^ ^]^ and epigenetic regulation of translation checkpoints and tumor progression involves the RUVBL1–EEF1A1 axis.^[^ ^47^ ^]^ In osteosarcoma, CircMYO10 promotes chromatin remodeling through RUVBL1 and enhances the transcriptional activity of the β‐catenin/LEF1 complex.^[^ ^56^ ^]^ In melanoma, RUVBL1 is involved in regulating chromatin remodeling to influence the transcriptional activity of β‐catenin.^[^ ^57^ ^]^ Through ATAC‐seq, we discovered that Lyn/RUVBL1 regulates the chromatin accessibility of β‐catenin. However, Lyn/RUVBL1 does not directly bind to the promoter region of CTNNB1 (which encodes β‐catenin). Pol II forms large clusters of aggregates in the nucleus of cells, which are often referred to as transcription factories or transcription condensates. Studies have shown that transcription factories can mediate the coordinated expression of genes at different chromatin sites, increase gene transcription efficiency, and organize 3D chromatin structures. RUVBL1 can directly control the POL II transcription factory located near the active promoter.^[^ ^38^ ^]^ In pancreatic cancer, RUVBL1 has been reported to occupy 10016 genomic loci, of which 7386 were located in the POL II promoter, and RUVBL1 depletion dramatically reduced the number of RUVBL1‐occupied loci to 805.^[^ ^58^ ^]^ HiCut is a technology that combines Hi‐C 3.0 and CUT‐Tag to obtain information about protein‐mediated spatial DNA interactions.^[^ ^59^ ^]^ HiCut showed that the binding ability of Pol II to TRIB3 was weakened after Lyn/RUVBL1 inhibition and that the interaction between the TRIB3 promoter and regulatory elements mediated by Pol II was weakened. In various tumors, such as colorectal cancer,^[^ ^39^ ^]^ lung cancer,^[^ ^40^ ^]^ and glioma,^[^ ^41^ ^]^ TRIB3 has been reported to physically interact with and activate β‐catenin. In endometrial cancer, TRIB3 may regulate CTNNB1 transcription by enhancing ELF4 recruitment to CTNNB1 promoters.^[^ ^60^ ^]^ In our study, we found that TRIB3 interacts with β‐catenin in colon cancer cells via Co‐IP. We suggest that TRIB3 regulates the expression of β‐catenin by interacting with β‐catenin.

Epigenetic remodeling directly affects tumor‐related metabolic disorders, thereby facilitating tumor progression. CHD6 recruits TCF4 to control the TMEM65 protein, which increases mitochondrial function and ATP production, leading to liver metastasis in CRC.^[^ ^61^ ^]^ After we knocked down Lyn, the differentially expressed genes identified via ATAC‐seq were enriched mainly in metabolic pathways, and the differentially expressed genes identified via HiCuT of Lyn/RUVBL1 were enriched mainly in fatty acid synthesis. It has been reported that inhibition of Lyn can block the endocytosis of CD36 and thus inhibit the intake of fatty acids.^[^ ^62^ ^]^ In addition, Lyn can regulate the metabolic reprogramming of free fatty acids to promote inflammation in steatohepatitis tissues.^[^ ^63^ ^]^ Hippocalcin‐like protein 1 inhibits liver lipid metabolism by directly targeting RUVBL1‐mTOR signal transduction to inhibit tumorigenicity.^[^ ^64^ ^]^ Metabolomics revealed that AA metabolism was inhibited in colon cancer cells after Lyn/RUVBL1 was knocked down. AA is the most widely distributed polyunsaturated fatty acid, which is consistent with the reported regulation of fatty acid metabolism by Lyn/RUVBL1. We observed decreased H3K27ac levels in the COX2 promoter and decreased chromatin accessibility of the transcription factor FOXA1. These results suggest that Lyn/RUVBL1 regulates AA through chromatin remodeling. On the other hand, we found that Lyn/RUVBL1 regulates FOXA1 expression via TRIB3. TRIB3 is closely related to ubiquitination. TRIB3 inhibited FOXO1 degradation by inhibiting FOXO1 ubiquitination.^[^ ^65^ ^]^ The E3 ligase complex formed by TRIB3 and TRIM8 can catalyze the K48 polyubiquitination of HNF4α and lead to its degradation.^[^ ^66^ ^]^ Therefore, we hypothesized that TRIB3 regulates FOXA1 expression by affecting FOXA1 ubiquitination. From the perspective of chromatin remodeling, our findings indicate that the Lyn/RUVBL1 complex modulates COX2, a pivotal enzyme in AA metabolism through FOXA1, and facilitates β‐catenin translocation into the nucleus to increase MMP9 and VEGF expression.

In conclusion, our findings indicate that a novel complex involving Lyn/RUVBL1 promotes the liver metastasis of CRC by regulating AA metabolism through epigenetic remodeling. This study may provide new research ideas for the precise treatment of CRC liver metastasis.

## 4 Experimental Section

### Proteomics of Co‐IP Mass Spectrometry Strip Identification

After the completion of Co‐IP, the protein was enzymatically degraded into polypeptide or peptide fragments. The total peptides extracted from each sample were subsequently separated via a nano‐UPLC liquid phase system known as EASY‐nLC1200. The resulting data were then collected via a QExactive HFX instrument equipped with a nanoliter ion source. Finally, the original data files were subjected to searching and analysis via Proteome Discoverer software (PD) (version 2.4.0.305; Thermo Fisher Scientific) along with its built‐in Sequest HT search engine (Biotree, China).

### GST Pull‐Down Assay

Protein–protein interactions in colon cancer cells were studied via a GST pull‐down kit (Thermo Fisher Scientific) according to the manufacturer's method. After the conversion of GST and GST‐Lyn (PPL, China) into BL21 (TransGen Biotech, China), IPTG (Macklin, China) was used to induce receptive cells. The GST‐tagged fusion protein was attached to a glutathione chromatographic column. The RUVBL1 overexpression plasmid was subsequently transformed into 293T cells. The proteins that interacted with the fusion proteins attached to the glutathione chromatographic column. Interacting proteins were eluted. The presence of binding proteins was analyzed by Western blotting.

### Proximity Ligation Assay (PLA)

Measurement of protein–protein interactions in colon cancer cells were performed according to the manufacturer's protocol using the NaveniFlex Cell MR Kit (Navinci, Sweden). In short, the fixed sample was enclosed in an incubator. The corresponding primary antibody was added for incubation. The PLA working mixture was then prepared for ligation and amplification. After the detection solution was added, the sample was washed and sealed to capture images under a fluorescence microscope.

### ATAC‐Seq

The library was constructed using Vazyme TD501 and TD202 kits (Vazyme, China). ATAC‐seq analysis was conducted in colon cancer cell lines through CRISPR/Cas9 knockdown and lentiviral knockdown of Lyn/RUVBL1 complex expression. The experimental procedures included sample quality assessment, DNA transposase fragmentation, purification of fragment products, PCR enrichment, library quality control, and computer sequencing. High‐quality valid data were filtered and compared with the reference genome for downstream genetic information analysis (Frasergen, China).

### RNA‐Seq

Lentivirus‐mediated knockdown of Lyn/RUVBL1 complex expression was performed in colon cancer cell lines, followed by total RNA extraction. Magnetic beads coated with oligo (dT) were utilized to enrich and purify the mRNA, which was then fragmented using a fragmentation buffer. The first strand of cDNA was synthesized from the short mRNA template using random primers. Subsequently, dNTPs, DNA polymerase I, RNaseH, and other synthetic cDNA strands were added to the system. The library was generated through elution, purification of double‐stranded cDNA, end repair, addition of base A and sequencing splicing. Finally, PCR amplification was conducted on the library. A quality assessment of colon cancer cell lines in which the Lyn/RUVBL1 complex was knocked down and control samples were performed before libraries were constructed for Illumina platform‐based sequencing (MetWare, China).

### Hi‐C Coupled Chromatin Cleavage and Tagmentation (HiCuT)

The Hi‐C 3.0 technique combined with CUT&Tag technology was employed to investigate the spatial interaction of DNA mediated by the target protein. The main experimental steps were as follows: the cell precipitate was collected and subjected to double crosslinking. Permeabilization of the cell membrane was achieved via the addition of 0.05% digitonin and 0.1% SDS, followed by neutralization of SDS with Triton X‐100. Chromatin DNA was subjected to double enzyme digestion (MboI+DdeI) via the Hi‐C 3.0 assay protocol, and subsequent repair of the DNA ends was performed using the Klenow enzyme. In situ ligations were carried out by using T4 DNA ligase to connect the fragments together. Following ligation, cells were further utilized in the CUT&Tag experiments. Magnetic beads coated with canavalin A were employed for cell binding. POLR2A antibody targeting the desired protein was added for incubation, followed by the addition of a secondary antibody for incubation. Protein G‐Tn5 (pG‐Tn5) fusion protein was then introduced, and unbound pG‐Tn5 molecules were washed away prior to the activation of Tn5 via the addition of Mg^2+^, enabling the transposition of binding sequences associated with the target protein. A Decrosslinking step ensued, followed by DNA extraction for PCR amplification, library construction, and high‐throughput sequencing (Frasergen, China).

### ChIP–qPCR

Chromatin immunoprecipitation (ChIP) experiments were performed using the SimpleChIP®Plus Enzymatic Chromatin IP Kit (Cell Signaling Technology, 9005). A total of 4 × 10^6^ cells were crosslinked to prepare the immunoprecipitation samples. After nuclear isolation and chromatin digestion, the lysates were centrifuged at 9400 × g at 4 °C for 10 min. The supernatant was diluted to a final volume of 500 µL with 1 × ChIP buffer containing a protease inhibitor, followed by the addition of a Polr2a‐targeting antibody (10 µL), incubation, elution, reversal of crosslinking, and purification. The target DNA fragments were subsequently collected. The analysis was performed via quantitative PCR and expressed as a percentage of the input DNA. The calculated result was obtained using the following formula: 2% × 2(C[T]2% input sample − C[T]IP sample). The primer sequences can be found in Table S4 (Supporting Information).

### Widely Targeted Metabolomics

The Lentiviral Lyn/RUVBL1 complex colon cancer cell line and its control group were subjected to knockdown to investigate changes in small molecule metabolites via widely targeted metabolomics. The metabolome differences among samples were investigated through multivariate statistical analysis, utilizing a self‐built database on an ultra‐performance liquid chromatography (UPLC) system (Shim‐pack UFLC SHIMADZU CBM30A) coupled with tandem mass spectrometry (MS/MS). The mass spectrum conditions included an electrospray ion source temperature of 500 °C, a positive ionization voltage of 5500 V, and a negative ionization voltage of −4500 V, as well as gas pressures for ion source gas I at 55 psi, gas II at 60 psi, and the curtain gas (CUR) at 25 psi (MetWare, China).

### Cleavage Under Targets and Tagmentation (CUT‐Tag)

Lyn/RUVBL1 knockout cells were harvested via in‐house Vazyme TD903 and TD202 kits (Vazyme, China). The samples were subsequently incubated with ConA beads, followed by incubation with H3K27ac antibody. A secondary antibody was then added for further incubation, followed by incubation with pA/G‐Tnp. Transposition was induced by the addition of magnesium ions, leading to the integration of the junction into nearby DNA. Gene amplification was subsequently performed, and the resulting PCR products were purified. Finally, Illumina PE150 library quality assessment and sequencing were conducted (Frasergen, China).

### Statistical Analysis

All the results are expressed as the means ± SDs. Statistical analysis was performed using SPSS 17.0 (SPSS) and GraphPad Prism 7. Student's *t*‐test, ANOVA, the log‐rank test, and the Pearson correlation coefficient were employed on the basis of the experimental design. A significance level of *p* < 0.05 was considered statistically significant. All patient tissues and clinical data were collected in accordance with the protocol approved by the Ethics Committee of the Second Hospital of Dalian Medical University, ensuring that written informed consent was obtained from each patient. All animal procedures followed the national Guidelines for the Care and Use of Laboratory Animals and received approval from the Animal Ethics Committee of Dalian Medical University.

## Conflict of Interest

The authors declare no conflict of interest.

## Author Contributions

S.R., Y.Z., and Z.Z. were responsible for the study concept and design. Z.Z., Y.G., Y.Q., B.W., K.J., Z.S., F.Z., M.Y., S.B., and X.Y. were responsible for performing the experiments, acquisition and analysis of data, and drafting the manuscript. S.R. provided and collected the clinical data. S.R. and Y.Z. were responsible for study supervision. S.R., Y.Z., and Z.Z. were responsible for the critical revision of the manuscript.

## Ethics Approval and Informed Consent

All patient tissues and clinical information were collected using protocols approved by the Ethics Committee of the Second Hospital of Dalian Medical University. Written informed consent was obtained from each patient. Ethics number is KY2024‐385‐01. All animal procedures used were approved by the Animal Ethics Committee of Dalian Medical University and conducted according to the national guidelines for the care and use of laboratory animals. Ethics number is AEE18083.

## Supporting information



Supporting Information

## Data Availability

The data that support the findings of this study are available from the corresponding author upon reasonable request.

## References

[1] H. Sung , J. Ferlay , R. L. Siegel , M. Laversanne , I. Soerjomataram , A. Jemal , F. Bray , CA Cancer J. Clin. 2021, 71, 209. 33538338 10.3322/caac.21660

[2] E. Dekker , P. J. Tanis , J. L. A. Vleugels , P. M. Kasi , M. B. Wallace , Lancet 2019, 394, 1467. 31631858 10.1016/S0140-6736(19)32319-0

[3] R. L. Siegel , A. N. Giaquinto , A. Jemal , CA Cancer J. Clin. 2024, 74, 12. 38230766 10.3322/caac.21820

[4] G. P. Nagaraju , B. Dariya , P. Kasa , S. Peela , B. F. El‐Rayes , Semin. Cancer Biol. 2022, 86, 622. 34324953 10.1016/j.semcancer.2021.07.017

[5] T. Heide , J. Househam , G. D. Cresswell , I. Spiteri , C. Lynn , M. Mossner , C. Kimberley , J. Fernandez‐Mateos , B. Chen , L. Zapata , C. James , I. Barozzi , K. Chkhaidze , D. Nichol , V. Gunasri , A. Berner , M. Schmidt , E. Lakatos , A. M. Baker , H. Costa , M. Mitchinson , R. Piazza , M. Jansen , G. Caravagna , D. Ramazzotti , D. Shibata , J. Bridgewater , M. Rodriguez‐Justo , L. Magnani , T. A. Graham , et al., Nature 2022, 611, 733. 36289335 10.1038/s41586-022-05202-1 PMC9684080

[6] W. R. Becker , S. A. Nevins , D. C. Chen , R. Chiu , A. M. Horning , T. K. Guha , R. Laquindanum , M. Mills , H. Chaib , U. Ladabaum , T. Longacre , J. Shen , E. D. Esplin , A. Kundaje , J. M. Ford , C. Curtis , M. P. Snyder , W. J. Greenleaf , Nat. Genet. 2022, 54, 985. 35726067 10.1038/s41588-022-01088-x PMC9279149

[7] V. Boonsanay , M. H. Mosa , M. Looso , D. Weichenhan , F. Ceteci , L. Pudelko , A. Lechel , C. S. Michel , C. Künne , H. F. Farin , C. Plass , F. R. Greten , Gastroenterology 2023, 164, 214. 36402192 10.1053/j.gastro.2022.10.036 PMC9889219

[8] A. Janney , F. Powrie , E. H. Mann , Nature 2020, 585, 509. 32968260 10.1038/s41586-020-2729-3

[9] H. Zhou , Q. Sun , M. Feng , Z. Gao , S. Jia , L. Cao , X. Yu , S. Gao , H. Wu , K. Li , Theranostics 2023, 13, 4247. 37554271 10.7150/thno.86528 PMC10405845

[10] N. Koundouros , E. Karali , A. Tripp , A. Valle , P. Inglese , N. J. S. Perry , D. J. Magee , S. Anjomani Virmouni , G. A. Elder , A. L. Tyson , M. L. Dória , A. van Weverwijk , R. F. Soares , C. M. Isacke , J. K. Nicholson , R. C. Glen , Z. Takats , G. Poulogiannis , Cell 2020, 181, 1596. 32559461 10.1016/j.cell.2020.05.053 PMC7339148

[11] Y. Yang , J. He , B. Zhang , Z. Zhang , G. Jia , S. Liu , T. Wu , X. He , N. Wang , Cell Death Dis. 2021, 12, 1108. 34839347 10.1038/s41419-021-04411-2 PMC8627508

[12] P. Bu , K. Y. Chen , K. Xiang , C. Johnson , S. B. Crown , N. Rakhilin , Y. Ai , L. Wang , R. Xi , I. Astapova , Y. Han , J. Li , B. B. Barth , M. Lu , Z. Gao , R. Mines , L. Zhang , M. Herman , D. Hsu , G. F. Zhang , X. Shen , Cell Metab. 2018, 27, 1249. 29706565 10.1016/j.cmet.2018.04.003 PMC5990465

[13] D. Hanahan , Cancer Discovery 2022, 12, 31. 35022204 10.1158/2159-8290.CD-21-1059

[14] Y. Wei , C. Tian , Y. Zhao , X. Liu , F. Liu , S. Li , Y. Chen , Y. Qiu , Z. Feng , L. Chen , T. Zhou , X. Ren , C. Feng , Y. Liu , W. Yu , H. Ying , Q. Ding , Nat. Metab. 2020, 2, 447. 32694659 10.1038/s42255-020-0203-z

[15] W. Wan , K. Peng , M. Li , L. Qin , Z. Tong , J. Yan , B. Shen , C. Yu , Oncogene 2017, 36, 3868. 28263974 10.1038/onc.2017.13

[16] O. G. McDonald , X. Li , T. Saunders , R. Tryggvadottir , S. J. Mentch , M. O. Warmoes , A. E. Word , A. Carrer , T. H. Salz , S. Natsume , K. M. Stauffer , A. Makohon‐Moore , Y. Zhong , H. Wu , K. E. Wellen , J. W. Locasale , C. A. Iacobuzio‐Donahue , A. P. Feinberg , Nat. Genet. 2017, 49, 367. 28092686 10.1038/ng.3753 PMC5695682

[17] M. Kim , J. Park , M. Bouhaddou , K. Kim , A. Rojc , M. Modak , M. Soucheray , M. J. McGregor , P. O'Leary , D. Wolf , E. Stevenson , T. K. Foo , D. Mitchell , K. A. Herrington , D. P. Muñoz , B. Tutuncuoglu , K. H. Chen , F. Zheng , J. F. Kreisberg , M. E. Diolaiti , J. D. Gordan , J. P. Coppé , D. L. Swaney , B. Xia , L. van ’t Veer , A. Ashworth , T. Ideker , N. J. Krogan , Science 2021, 374, eabf3066. 34591612 10.1126/science.abf3066 PMC9040556

[18] D. L. Swaney , D. J. Ramms , Z. Wang , J. Park , Y. Goto , M. Soucheray , N. Bhola , K. Kim , F. Zheng , Y. Zeng , M. McGregor , K. A. Herrington , R. O'Keefe , N. Jin , N. K. VanLandingham , H. Foussard , J. Von Dollen , M. Bouhaddou , D. Jimenez‐Morales , K. Obernier , J. F. Kreisberg , M. Kim , D. E. Johnson , N. Jura , J. R. Grandis , J. S. Gutkind , T. Ideker , N. J. Krogan , Science 2021, 374, eabf2911. 34591642 10.1126/science.abf2911 PMC9005332

[19] F. Zheng , M. R. Kelly , D. J. Ramms , M. L. Heintschel , K. Tao , B. Tutuncuoglu , J. J. Lee , K. Ono , H. Foussard , M. Chen , K. A. Herrington , E. Silva , S. N. Liu , J. Chen , C. Churas , N. Wilson , A. Kratz , R. T. Pillich , D. N. Patel , J. Park , B. Kuenzi , M. K. Yu , K. Licon , D. Pratt , J. F. Kreisberg , M. Kim , D. L. Swaney , X. Nan , S. I. Fraley , J. S. Gutkind , et al., Science. 2021, 374, eabf3067. 34591613 10.1126/science.abf3067 PMC9126298

[20] W. Dong , A. Fekete , X. Chen , H. Liu , G. L. Beilhartz , X. Chen , S. Bahrampour , Y. Xiong , Q. Yang , H. Zhao , T. Kong , M. S. Morioka , G. Jung , J. E. Kim , D. Schramek , P. B. Dirks , Y. Song , T. H. Kim , Y. He , S. Wanggou , X. Li , R. A. Melnyk , L. Y. Wang , X. Huang , Nat. Cancer 2023, 4, 1418. 37697045 10.1038/s43018-023-00626-8

[21] B. Yao , T. Gui , X. Zeng , Y. Deng , Z. Wang , Y. Wang , D. Yang , Q. Li , P. Xu , R. Hu , X. Li , B. Chen , J. Wang , K. Zen , H. Li , M. J. Davis , M. J. Herold , H. F. Pan , Z. W. Jiang , D. C. S. Huang , M. Liu , J. Ju , Q. Zhao , Genome Med. 2021, 13, 58. 33853662 10.1186/s13073-021-00871-5 PMC8048298

[22] W. Wang , Y. A. Tang , Q. Xiao , W. C. Lee , B. Cheng , Z. Niu , G. Oguz , M. Feng , P. L. Lee , B. Li , Z. H. Yang , Y. F. Chen , P. Lan , X. J. Wu , Q. Yu , Nat. Commun. 2021, 12, 4441. 34290255 10.1038/s41467-021-24687-4 PMC8295257

[23] E. Ingley , Cell Commun. Signal. 2012, 10, 21. 22805580 10.1186/1478-811X-10-21 PMC3464935

[24] L. Fattet , H. Y. Jung , M. W. Matsumoto , B. E. Aubol , A. Kumar , J. A. Adams , A. C. Chen , R. L. Sah , A. J. Engler , E. B. Pasquale , J. Yang , Dev. Cell 2020, 54, 302. 32574556 10.1016/j.devcel.2020.05.031 PMC7423770

[25] N. Su , L. Peng , B. Xia , Y. Zhao , A. Xu , J. Wang , X. Wang , B. Jiang , Mol. Cancer 2012, 11, 43. 22731636 10.1186/1476-4598-11-43 PMC3464950

[26] T. Powles , J. Brown , J. Larkin , R. Jones , C. Ralph , R. Hawkins , S. Chowdhury , E. Boleti , A. Bhal , K. Fife , A. Webb , S. Crabb , T. Geldart , R. Hill , J. Dunlop , P. E. Hall , D. McLaren , C. Ackerman , L. Beltran , P. Nathan , Ann. Oncol. 2016, 27, 880. 26802156 10.1093/annonc/mdw014

[27] L. Lang , C. Shay , Y. Xiong , P. Thakkar , R. Chemmalakuzhy , X. Wang , Y. Teng , J. Hematol. Oncol. 2018, 11, 85. 29925404 10.1186/s13045-018-0623-3 PMC6011403

[28] J. J. Arcaroli , B. M. Touban , A. C. Tan , M. Varella‐Garcia , R. W. Powell , S. G. Eckhardt , P. Elvin , D. Gao , W. A. Messersmith , Clin. Cancer Res. 2010, 16, 4165. 20682712 10.1158/1078-0432.CCR-10-0066 PMC3805460

[29] J. Tang , Y. Xiao , G. Lin , H. Guo , H. X. Deng , S. Tu , W. Y. Langdon , H. Yang , L. Tao , Y. Li , R. M. Pope , N. Gupta , J. Zhang , Sci. Signal. 2021, 14, eabe3410. 34699250 10.1126/scisignal.abe3410 PMC8815314

[30] T. Ban , G. R. Sato , A. Nishiyama , A. Akiyama , M. Takasuna , M. Umehara , S. Suzuki , M. Ichino , S. Matsunaga , A. Kimura , Y. Kimura , H. Yanai , S. Miyashita , J. Kuromitsu , K. Tsukahara , K. Yoshimatsu , I. Endo , T. Yamamoto , H. Hirano , A. Ryo , T. Taniguchi , T. Tamura , Immunity 2016, 45, 319. 27521268 10.1016/j.immuni.2016.07.015

[31] C. Tang , M. Ke , X. Yu , S. Sun , X. Luo , X. Liu , Y. Zhou , Z. Wang , X. Cui , C. Gu , Y. Yang , Adv. Sci. (Weinh) 2023, 10, e2301264. 37439412 10.1002/advs.202301264 PMC10477903

[32] T. P. Green , M. Fennell , R. Whittaker , J. Curwen , V. Jacobs , J. Allen , A. Logie , J. Hargreaves , D. M. Hickinson , R. W. Wilkinson , P. Elvin , B. Boyer , N. Carragher , P. A. Plé , A. Bermingham , G. A. Holdgate , W. H. Ward , L. F. Hennequin , B. R. Davies , G. F. Costello , Mol. Oncol. 2009, 3, 248. 19393585 10.1016/j.molonc.2009.01.002 PMC5527863

[33] V. A. Assimon , Y. Tang , J. D. Vargas , G. J. Lee , Z. Y. Wu , K. Lou , B. Yao , M. K. Menon , A. Pios , K. C. Perez , A. Madriaga , P. K. Buchowiecki , M. Rolfe , L. Shawver , X. Jiao , R. Le Moigne , H. J. Zhou , D. J. Anderson , ACS Chem. Biol. 2019, 14, 236. 30640450 10.1021/acschembio.8b00904

[34] K. Jiang , H. Chen , Y. Fang , L. Chen , C. Zhong , T. Bu , S. Dai , X. Pan , D. Fu , Y. Qian , J. Wei , K. Ding , J. Exp. Clin. Cancer Res. 2021, 40, 21. 33413536 10.1186/s13046-020-01816-3 PMC7792106

[35] B. Zhang , S. K. Halder , N. D. Kashikar , Y. J. Cho , A. Datta , D. L. Gorden , P. K. Datta , Gastroenterology 2010, 138, 969. 19909744 10.1053/j.gastro.2009.11.004 PMC2831103

[36] K. Ohashi , Z. Wang , Y. M. Yang , S. Billet , W. Tu , M. Pimienta , S. L. Cassel , S. J. Pandol , S. C. Lu , F. S. Sutterwala , N. Bhowmick , E. Seki , Hepatology 2019, 70, 1582. 31044438 10.1002/hep.30693 PMC6819206

[37] M. Yuan , X. Zhang , J. Zhang , K. Wang , Y. Zhang , W. Shang , Y. Zhang , J. Cui , X. Shi , H. Na , D. Fang , Y. Zuo , S. Ren , Cell Death Differ. 2020, 27, 379. 31217502 10.1038/s41418-019-0361-2 PMC7205996

[38] H. Wang , B. Li , L. Zuo , B. Wang , Y. Yan , K. Tian , R. Zhou , C. Wang , X. Chen , Y. Jiang , H. Zheng , F. Qin , B. Zhang , Y. Yu , C. P. Liu , Y. Xu , J. Gao , Z. Qi , W. Deng , X. Ji , Nat. Commun. 2022, 13, 5703. 36171202 10.1038/s41467-022-33433-3 PMC9519968

[39] F. Hua , S. Shang , Y. W. Yang , H. Z. Zhang , T. L. Xu , J. J. Yu , D. D. Zhou , B. Cui , K. Li , X. X. Lv , X. W. Zhang , S. S. Liu , J. M. Yu , F. Wang , C. Zhang , B. Huang , Z. W. Hu , Gastroenterology 2019, 156, 708. 30365932 10.1053/j.gastro.2018.10.031

[40] X. Zhang , N. Zhong , X. Li , M. B. Chen , Eur. J. Pharmacol. 2019, 863, 172697. 31562867 10.1016/j.ejphar.2019.172697

[41] Y. Lu , L. Li , L. Chen , Y. Gao , X. Chen , Y. Cao , Environ. Toxicol. 2020, 35, 697. 31995275 10.1002/tox.22905

[42] L. Sun , C. Suo , T. Zhang , S. Shen , X. Gu , S. Qiu , P. Zhang , H. Wei , W. Ma , R. Yan , R. Chen , W. Jia , J. Cao , H. Zhang , P. Gao , Nat. Chem. Biol. 2023, 19, 1492. 37500770 10.1038/s41589-023-01391-6

[43] J. S. Runge , J. R. Raab , T. Magnuson , G3 (Bethesda) 2018, 8, 1095. 29432129 10.1534/g3.117.300504 PMC5873900

[44] K. W. Brudvik , J. E. Paulsen , E. M. Aandahl , B. Roald , K. Taskén , Mol. Cancer 2011, 10, 149. 22168384 10.1186/1476-4598-10-149 PMC3278393

[45] T. Cho , R. Romagnuolo , C. Scipione , M. B. Boffa , M. L. Koschinsky , Mol. Biol. Cell 2013, 24, 210. 23243000 10.1091/mbc.E12-08-0637 PMC3564524

[46] M. A. Kamel , J. L. Picconi , N. Lara‐Castillo , M. L. Johnson , Bone 2010, 47, 872. 20713195 10.1016/j.bone.2010.08.007 PMC2952691

[47] M. Li , L. Yang , A. K. N. Chan , S. P. Pokharel , Q. Liu , N. Mattson , X. Xu , W. H. Chang , K. Miyashita , P. Singh , L. Zhang , M. Li , J. Wu , J. Wang , B. Chen , L. N. Chan , J. Lee , X. H. Zhang , S. T. Rosen , M. Müschen , J. Qi , J. Chen , K. Hiom , A. J. R. Bishop , C. W. Chen , Adv. Sci. (Weinh) 2023, 10, e2206584. 37075745 10.1002/advs.202206584 PMC10265057

[48] M. Kanemaki , Y. Makino , T. Yoshida , T. Kishimoto , A. Koga , K. Yamamoto , M. Yamamoto , V. Moncollin , J. M. Egly , M. Muramatsu , T. Tamura , Biochem. Biophys. Res. Commun. 1997, 235, 64. 9196036 10.1006/bbrc.1997.6729

[49] X. Liu , J. Chen , S. Zhang , X. Liu , X. Long , J. Lan , M. Zhou , L. Zheng , J. Zhou , EMBO Rep. 2022, 23, e54128. 35876654 10.15252/embr.202154128 PMC9442307

[50] Y. Xu , K. W. Harder , N. D. Huntington , M. L. Hibbs , D. M Tarlinton , Immunity 2005, 22, 9. 15664155 10.1016/j.immuni.2004.12.004

[51] S. Sipeki , K. Koprivanacz , T. Takács , A. Kurilla , L. László , V. Vas , L. Buday , Cells 2021, 10, 1191. 34068055 10.3390/cells10051191 PMC8152464

[52] M. Dülk , B. Szeder , G. Glatz , B. L. Merő , K. Koprivanacz , G. Kudlik , V. Vas , S. Sipeki , A. Cserkaszky , L. Radnai , L. Buday , Biochemistry 2018, 57, 4186. 29928795 10.1021/acs.biochem.8b00084

[53] S. Hammond , A. Wagenknecht‐Wiesner , S. L. Veatch , D. Holowka , B. Baird , J. Struct. Biol. 2009, 168, 161. 19427382 10.1016/j.jsb.2009.04.012 PMC2767321

[54] S. Kubota , M. Morii , R. Yuki , N. Yamaguchi , H. Yamaguchi , K. Aoyama , T. Kuga , T. Tomonaga , N. Yamaguchi , J. Biol. Chem. 2015, 290, 10891. 25770215 10.1074/jbc.M115.643882 PMC4409252

[55] R. Toth , D. Scherer , L. E. Kelemen , A. Risch , A. Hazra , Y. Balavarca , J. J. Issa , V. Moreno , R. A. Eeles , S. Ogino , X. Wu , Y. Ye , R. J. Hung , E. L. Goode , C. M. Ulrich , Cancer Epidemiol. Biomarkers Prev. 2017, 26, 816. 28115406 10.1158/1055-9965.EPI-16-0728 PMC6054308

[56] J. Chen , G. Liu , Y. Wu , J. Ma , H. Wu , Z. Xie , S. Chen , Y. Yang , S. Wang , P. Shen , Y. Fang , S. Fan , S. Shen , X. Fang , Mol. Cancer 2019, 18, 150. 31665067 10.1186/s12943-019-1076-1 PMC6819556

[57] C. Zhang , S. Wu , Cell Death Discovery 2023, 9, 132. 37076452 10.1038/s41420-023-01429-7 PMC10115834

[58] M. Vogt , N. Dudvarski Stankovic , Y. Cruz Garcia , J. Hofstetter , K. Schneider , F. Kuybu , T. Hauck , B. Adhikari , A. Hamann , Y. Rocca , L. Grysczyk , B. Martin , A. Gebhardt‐Wolf , A. Wiegering , M. Diefenbacher , G. Gasteiger , S. Knapp , D. Saur , M. Eilers , M. Rosenfeldt , F. Erhard , S. M. Vos , E. Wolf , Gut 2024, 73, 1509. 38821858 10.1136/gutjnl-2023-331519 PMC11347226

[59] S. Sati , P. Jones , H. S. Kim , L. A. Zhou , E. Rapp‐Reyes , T. H. Leung , PLoS Genet. 2022, 18, e1010121. 35320278 10.1371/journal.pgen.1010121 PMC8979432

[60] W. L. Wang , G. C. Hong , P. J. Chien , Y. H. Huang , H. T. Lee , P. H. Wang , Y. C. Lee , W. W. Chang , Cancers (Basel) 2020, 12, 3785. 33334065 10.3390/cancers12123785 PMC7765506

[61] B. Zhang , Q. Liu , W. Wen , H. Gao , W. Wei , A. Tang , B. Qin , H. Lyu , X. Meng , K. Li , H. Jin , F. Yu , Q. Pan , J. Lin , M. H. Lee , Cell Discovery 2022, 8, 130. 36473865 10.1038/s41421-022-00478-z PMC9727023

[62] J. W. Hao , J. Wang , H. Guo , Y. Y. Zhao , H. H. Sun , Y. F. Li , X. Y. Lai , N. Zhao , X. Wang , C. Xie , L. Hong , X. Huang , H. R. Wang , C. B. Li , B. Liang , S. Chen , T. J. Zhao , Nat. Commun. 2020, 11, 4765. 32958780 10.1038/s41467-020-18565-8 PMC7505845

[63] L. Zhao , C. Zhang , X. Luo , P. Wang , W. Zhou , S. Zhong , Y. Xie , Y. Jiang , P. Yang , R. Tang , Q. Pan , A. R. Hall , T. V. Luong , J. Fan , Z. Varghese , J. F. Moorhead , M. Pinzani , Y. Chen , X. Z. Ruan , J. Hepatol. 2018, 69, 705. 29705240 10.1016/j.jhep.2018.04.006

[64] T. Chen , Z. Yuan , Z. Lei , J. Duan , J. Xue , T. Lu , G. Yan , L. Zhang , Y. Liu , Q. Li , Y. Zhang , Theranostics 2022, 12, 7450. 36438486 10.7150/thno.75936 PMC9691343

[65] J. M. Yu , W. Sun , Z. H. Wang , X. Liang , F. Hua , K. Li , X. X. Lv , X. W. Zhang , Y. Y. Liu , J. J. Yu , S. S. Liu , S. Shang , F. Wang , Z. N. Yang , C. X. Zhao , X. Y. Hou , P. P. Li , B. Huang , B. Cui , Z. W. Hu , Nat. Commun. 2019, 10, 5720. 31844113 10.1038/s41467-019-13700-6 PMC6915745

[66] M. C. Xiao , N. Jiang , L. L. Chen , F. Liu , S. Q. Liu , C. H. Ding , S. H. Wu , K. Q. Wang , Y. Y. Luo , Y. Peng , F. Z. Yan , X. Zhang , H. Qian , W. F. Xie , J. Hepatol. 2024, 80, 778. 38237865 10.1016/j.jhep.2023.12.029

