# Supplementary material for: The Lyn/RUVBL1 Complex Promotes Colorectal Cancer Liver Metastasis by Regulating Arachidonic Acid Metabolism Through Chromatin Remodeling
Source: Adv Sci (Weinh). 2024 Dec 12;12(5):2406562. doi: 10.1002/advs.202406562 (PMC11792055; doi:10.1002/advs.202406562)
Supplement: Supplementary file 1 — Supporting Information [file ADVS-12-2406562-s001.docx]

**The Lyn/RUVBL1 Complex Promotes Colorectal Cancer Liver Metastasis by Regulating Arachidonic Acid Metabolism through Chromatin Remodeling**

Zhenyu Zhang^2^; Yina Gao^2^; Yuanyuan Qian^2^; Bowen Wei^2^; Kexin Jiang^2^; Zhiwei Sun^1^; Feifan Zhang^1^; Mingming Yang^2^; Salem Baldi^2^; Xiaoqi Yu^2^; Yunfei Zuo^2^*; Shuangyi Ren^1^*

**Author Affiliations:**

^1^Department of General Surgery, The Second Hospital of Dalian Medical University, Dalian 116023, China.

^2^Department of Clinical Biochemistry, College of Laboratory Diagnostic Medicine, Dalian Medical University, Dalian 116044, China.

**This file includes:**

**Supplementary Materials and Methods**

**Supplementary Tables**

**Supplementary Figures and Figure legends**

**Supplementary Materials and Methods**

**Cell culture:** Human colon cancer cells (HCT116, LS174T and LoVo) and human embryonic kidney cells (HEK-293T) were purchased from the Institute of Biochemistry and Cell Biology, Chinese Academy of Sciences (Shanghai, China). Human normal hepatocytes (THLE-2) were purchased from Pricella (Wuhan, China), and the cells were cultured in THLE-2-specific culture medium. The cells were cultured in DMEM or RPMI-1640 (Gibco, USA) medium supplemented with 10% fetal bovine serum (FBS, NEWZERUM, NZL). All the cell lines were placed in a 5% CO_2_ incubator at 37 °C and maintained at a certain humidity.

**Plasmid construction, siRNA treatment, and lentiviral transfection:** sgRNA sequences were designed and synthesized using the CRISPick website and then linked to the BBSI-treated px330a dCas9-KRAB vector after annealing. The shRNA sequence was designed and synthesized by Sigma, annealed and connected in the pLKO.1 TRC vector. The target DNA was added to the receptive cells, and after transformation, they were evenly coated on solid media supplemented with antibiotics and cultured overnight at 37 °C. The monoclonal colonies were selected in liquid medium for sequencing, and the plasmids and packaging plasmids were cotransfected into receptive cells. The lentivirus suspension was collected 72 h later. The RUVBL1 and FOXA1 overexpression plasmids were purchased from PPL (Nanjing, China). All small interfering RNAs (siRNAs) were synthesized by GenePharma to knock down the corresponding molecules in colon cancer cells. siRNAs were transfected into cells via the GP-transfect-Mate transfection reagent (GenePharma, Suzhou, China) according to the manufacturer's instructions. mRNA was collected 24–48 h after transfection, and protein was collected 48–72 h after transfection. The CRISPR-Lyn lentivirus, CRISPR-RUVBL1 lentivirus, FOXA1 knockdown lentivirus and negative control lentivirus were produced by GenePharma, and 1–10 ng/ml puromycin was used to screen stable knockdown cell lines. All sequences are listed in Supplementary Table S2.

**RNA extraction and PCR detection:** Total RNA was extracted from cell lines and colon cancer tissues using a TRIzol kit (Accurate Biology), and reverse transcription was performed using HiScript II Reverse Transcriptase (Vazyme Biotech). Then, a polymerase chain reaction was performed. The primers used were purchased from Sangon (China). All primers used are listed in Supplementary Table S3. Cycle threshold (Ct) values were used to determine the expression levels of each gene, and the relative expression levels were calculated via the 2^−ΔΔCt^ method and normalized to the endogenous reference gene GAPDH.

**Western blotting:** The total protein was extracted using a total protein extraction kit (Solarbio, China), and the protein concentration was measured using a BCA protein concentration assay kit (Sevenbio, China). The boiled protein samples were subjected to SDS‒PAGE, transferred to cellulose nitrate film, blocked with 5% skim milk powder at room temperature for 2 h, incubated with primary antibody at 4 °C overnight, incubated with the corresponding secondary antibody at room temperature for 2 h, and finally developed and preserved in a chemiluminescence imaging device. All the antibodies used are listed in Supplementary Table S5.

**Immunohistochemistry:** Tissue sections were dewaxed in a xylene solution, followed by antigen retrieval through boiling in citrate buffer and natural cooling to room temperature. Peroxidase removal was performed with 3% H_2_O_2_. The tissue was subsequently sealed with goat serum at room temperature for 30 minutes. The membranes were incubated with primary antibody overnight at 4 °C, followed by incubation with the corresponding secondary antibody at room temperature for 2 hours. Finally, fresh DAB solution was added for staining, and the tissue sections were restained, dehydrated, made transparent, and sealed.

**Co-Immunoprecipitation (Co-IP):** A Co-IP assay was used for precipitation (Thermo Fisher Scientific, USA). Lyn SH2, SH3, SH2+3, K418R, and His-Ub plasmids were purchased from PPL. The cell precipitates were collected and added to the immunoprecipitation lysate, which was cleaved on ice for 30 min. Pierce protein A/G magnetic beads were crosslinked with DSS for 30 min, and the resulting cell lysates were incubated with magnetic beads at 4 °C overnight. The magnetic beads were cleaned with immunoprecipitation lysis/rinsing buffers and ultrapure water to eluate the bound antigens. The supernatant was then boiled for subsequent SDS‒PAGE and western blot analysis.

**Silver staining assay:** The protein samples were fixed with fixative solution via Western blot electrophoresis. The experiment was conducted using the Fast Silver Stain Kit (Beyotime, China). The samples were subsequently washed with ethanol and water before they were treated with silver dye sensitizing solution. Following another round of water washing, silver solution was added. The mixture was then shaken at room temperature for 10 minutes. When the desired bands became visible, the solution was promptly discarded and replaced with silver dye stopping solution.

**Counting Kit-8 assay:** The cells were inoculated into a 96-well plate at a density of 2×10^3^ per well, with each group containing 3 wells. On Days 0, 1, 2, and 3 after inoculation, 10 µL of CCK-8 solution was added to each well of the 96-well plate and incubated for 4 hours in the dark (APExBIO, USA). Following termination of the cell culture, enzymatic hydrolysis was used to measure the absorbance of each well at a wavelength of 450 nm.

**Immunofluorescence:** The cells were fixed in a 4% paraformaldehyde solution, permeabilized with 0.5% Triton X-100, blocked with goat serum at room temperature for 30 minutes, incubated overnight at 4 °C with the primary antibody, and then incubated with the corresponding secondary antibody in the dark at room temperature for 2 hours. Finally, DAPI (Solarbio) was added, and the samples were incubated in the dark for 10–15 minutes. Images were observed and saved using a fluorescence microscope as soon as possible.

**Transwell assay:** Variou treated colon cancer cells were added to a chamber with an 8 μm pore size. Saracatinib and PGE2 were purchased from MCE. CB-6644 was obtained from Sunshine (Wuhan, China). The solutions were added to the cells according to the concentrations reported in the study. CB - 6644 (0.4 μM) ^[32]^, saracatinib (0.22 μM) ^[33]^, and PGE2 (10 μM) ^[42]^. The cell suspension, which was resuspended in serum-free medium, was added to the upper compartment of the chamber, and serum-containing medium was added to the lower compartment. After 24 hours, the chamber was removed, and the cells were fixed in a 4% paraformaldehyde solution for 30 minutes, followed by staining with 0.5% crystal violet for 15 minutes. The excess crystal violet on the upper side of the chamber was subsequently gently removed via a cotton swab after cleaning with PBS solution. Finally, the chamber was air-dried and imaged using a microscope for analysis via ImageJ software.

**Coculture experiment:** Colon cancer cells were suspended in serum-free medium and cultured above the chamber. When the colon cancer cells were attached to the wall, the medium was replaced with medium containing 0.5% serum. The medium of human liver cells was also replaced with medium containing 0.5% serum, and 24-well plates were established. After the hepatocytes were attached to the wall, the cells were placed above the 24-well plate for coculture. After 24 hours, the chamber was removed, and the cells were fixed in a solution of 4% paraformaldehyde for 30 minutes before they were stained with 0.5% crystal violet for an additional 15 minutes. Nonmigrating cells on the upper side of the chamber were gently removed via cotton swabs, dried, and then photographed under a microscope. Finally, ImageJ software was used to analyze and interpret the experimental results.

**Nude mouse model of liver metastasis in CRC:** A total of 136 male and female nude mice were purchased from Beijing Vital River (China). Stable knockdown of Lyn and RUVBL1 in colon cancer cells was achieved by using lentiviral vectors and CRISPR/Cas9. Four experimental groups were established: the Lyn-knockdown group, the RUVBL1-knockdown group, the Lyn and RUVBL1-co-knockdown group, and the control group. Nude mice were anesthetized with tribromoethanol, followed by a surgical procedure to inject colon cancer cells into the liver or spleen. Tumor growth and invasion were monitored via a bioluminescent imaging system to detect fluorescent signals in the nude mice. The survival days of the mice were recorded until they reached a near-death state due to tumor progression. Survival curves were generated for each experimental group. After the mice were sacrificed, their spleens and livers were excised for further analysis, including photographic documentation. Paraffin sections of these tissues were fixed with 4% paraformaldehyde for H&E staining to observe tumor metastasis.

**Hematoxylin–Eosin Staining (HE):** The paraffin sections were melted and immersed in a xylene solution followed by gradient alcohol for dewaxing and dehydration. Hematoxylin was used to stain the cell nucleus, while excess blue color on the cytoplasm was eliminated by adding 1% hydrochloric acid alcohol. Eosin was subsequently used to stain the cytoplasm, which was then dehydrated with gradient alcohol and xylene. Finally, the slices were cut for natural air drying before they were carefully sealed with neutral resin to ensure bubble-free processing. After drying and solidification of the neutral resin, microscopic observation was conducted, and images were collected for subsequent analysis.

**Plate clone assay:** The cells were inoculated at a density of 2×10^3^ per well in 6-well plates and cultured under 5% CO_2_ at 37 °C. After incubation for 14 days, the medium was removed, and the cells were washed twice with PBS before they were fixed with 4% paraformaldehyde for 15 minutes. Subsequently, the colonies were stained with a solution of crystal violet (0.5%, w/v; 5 μg/mL), followed by two washes with PBS. Colonies containing more than fifty cells were counted after drying overnight. The experiment was repeated three times to obtain an average value.

**Enzyme-linked immunosorbent assay (ELISA):** Experiments were conducted using ELISA kits (Elabscience, China). Standard wells, blank wells, and sample wells were prepared. A double dilution standard was added to the standard wells, while a mixture of standard and sample diluents was added to the blank wells. The test samples were added to the remaining wells. Biotinylated antibody working solution was then added to each well. The enzyme-labeled plates were coated and incubated at 37 °C for 45 minutes. The mixture was removed from the plate and washed with washing solution. An enzyme conjugate working solution was added to each well, the mixture was covered with a film, and it was incubated at 37 °C for 30 minutes. The liquid was discarded, and the samples were washed again with washing solution. Substrate solution was added to each well, and the samples were covered with film and incubated at 37 °C for 15 minutes in darkness. The reaction was stopped by adding a termination solution. Finally, the optical density of each well was measured immediately via an enzyme labeler at a wavelength of 450 nm.

**Dual-luciferase reporter assay:** The dual-luciferase reporter gene plasmid was obtained from GENEWIZ, and the plasmid along with FOXA1 siRNA was cotransfected into colon cancer cells. The cells were subsequently lysed, and the luciferase assay working solution was added. The determination of the relative light unit (RLU) was carried out using a multifunctional enzyme marker at 2-second intervals for a total duration of 10 seconds. Firefly luciferase detection reagent was introduced and mixed to determine the RLU values. Similarly, RLU values were determined by adding and mixing the luciferase assay working mixture. In cases involving sea luciferase, the RLU value determined by firefly luciferase was divided by that determined by sea luciferase to obtain a ratio that facilitated the comparison of target reporter gene activation levels across different samples (Beyotime, China).

**Scratch assay:** On the reverse side of the 6-well plate, a marker was used to delineate three horizontal lines parallel to the ruler. The cells were inoculated into 6-well plates and cultured conventionally. Upon reaching a density of 90–100%, they were substituted with serum-free medium via a sterile 200 µL pipette tip inserted perpendicularly to the bottom surface. Subsequently, under an inverted microscope, the scratch was observed and documented at 0, 12, and 24 hours. The percentage of the scratched area was quantified via ImageJ software. The experiment was repeated three times, after which the average was calculated.

**Database analysis:** The Ualcan ([https://ualcan.path.uab.edu/](https://ualcan.path.uab.edu/index.html) ) and GEPIA2 (http://gepia2.cancer-pku.cn) databases were utilized for the identification of proteins exhibiting high expression in colon cancer. Survival analysis of colon cancer patients using Kaplan-Meier plotter (https://kmplot.com/analysis/). Differential gene analysis of ATAC-seq and RNA-seq data was conducted using the DAVID (https://david.ncifcrf.gov/) for KEGG and GO pathway enrichment analysis. Additionally, differential metabolite analysis was performed using MetaboAnalyst 6.0 (https://www.metaboanalyst.ca/) for KEGG pathway analysis. COX2 transcription factors were searched for utilizing the hTFtarget (https://guolab.wchscu.cn/hTFtarget/) and AnimalTFDB (https://guolab.wchscu.cn/AnimalTFDB4/) databases.

**Supplementary Table S1. Proteins in Co-IP that specifically interact with Ip and not with IgG**

| GENE | | SCORE | | IgG | Ip |  |
| --- | --- | --- | --- | --- | --- | --- |
| PRRC2A  PTPN23  NCOR1  ARID1A  TBL1XR1  SCYL1  SNRNP200  FIP1L1  DDX3X  RUVBL1  ZYX  IGKV3-15  ANK3  CPSF1  MED4  LDHC  CTTNBP2NL  TUT4  ATP5F1B  HRNR  CCT3  EIF3I  PAPOLG  SPRR1B  KHNYN  HNRNPK  RPS4X  CKAP4 | | 16.63  9.96  6.9  5.98  5.47  5.04  4.58  3.86  3.06  2.96  2.79  2.54  2.54  2.54  2.45  2.43  2.33  2.26  2.17  2.16  2.11  2.05  2.04  2.02  1.97  1.94  1.93  1.9 | |  | 946072.1563  1455617.469  639864.3438  1096259.523  411995.625  409919.75  256233.9922  88757.375  157498.8125  426238.7813  290596.625  136745.5625  222437.4531  137677.7969  286941.25  174930.9531  147471.6563  287234.9063  175059.3281  118007.7734  109134.3125  99927.27344  141962.6875  236737.6406  158911.4531  205675.2344  1368671.25  187525.0469 |  |
| TJP2 | 1.9 | |  | | 350555.9063 | |

**Supplementary Table S2. shRNA or siRNA sequence**

| Name Sequence (5' to 3') |
| --- |
| Lyn shRNA GAGTGACGATGGAGTAGATTT  RUVBL1 shRNA CCACAGAATTCGACCTTGATT  FOXA1 shRNA1 GGACTTCAAGGCATACGAA  FOXA1 shRNA2 GAACTCCATGAACACCTACAT  Control shRNA GTTCTCCGAACGTGTCACGT  Scr siRNA UUCUCCGAACGUGUCACGUTT  Lyn siRNA GCAUGGAGAAUGGUGGAAATT  RUVBL1 siRNA CCACAGAAUUCGACCUUGATT  TRIB3 siRNA GGACCUGAGAUACUCAGCUTT  COX2 siRNA GCUGGGAAGCCUUCUCUAATT  FOXA1 siRNA GGACUUCAAGGCAUACGAATT  Cas9-Lyn#1 CACCGAGGCTGCAGCGCGTCTGGCC  Cas9-Lyn#2 CACCGCTGCAGCGCGTCTGGCCCGG  CRISPR Lyn gRNA1-1 CCGGGTGGATGCCATCATAG  CRISPR Lyn gRNA1-2 AGGAGAGAAGATGAAAGTCC  CRISPR Lyn gRNA2-1 ACAATGTCTCCTTGTTCCTC  CRISPR Lyn gRNA2-2 GTAGCCTTGTACCCCTATGA |

**Supplementary Table S3. Quantitative RT-PCR primers**

| Gene name Forward (5’to3’) Reverse(5’to3’) |
| --- |
| Lyn TGTGAGAGATCCAACGTCCA GAAAGACAAGTCGTCCGGGT  RUVBL1 AAAGAGCGAGTAGAAGCTGGA CCAAGTCATGCAAGGTCACATC  CCT3 ATTGGCCCGCATAAGGTAGT ACGAGGGTGGGTGATTTGTT  CKAP4 TGTGAAGATGGCGATGTTGT CAGCCGGATCAGCGAAGT  RPS4X AGATTTGCATGCAGCGGTTC GGCCTCCTCAGGTGTAATACG  EIF3I CTCAAGACCAATTCGGCTGTC CTGGTAGCCCATCTGCTTGTC  TJP2 GACGTCAATCGTCATCTCAGATGT GCTGCGGAAACTTCTGCCATCAAA  CTNNB1 TTTAAGCCTCTCGGTCTGTG CAAATACCCTCAGGGGAACA  TNS4 TGTTTGGAAGCAATCAATCAGTCCCT TACTAGGAGCCTGGGCATCA  GDF15 CTCCAGATTCCGAGAGTTGC AGAGATACGCAGGTGCAGG  FOXQ1 GTGATTTCTTGCTATTGACCGATG GCCCAAGGAGACCACAGTTAGA  SOX9 GCACATCAAGACGGAGCAG GTAGGTGAAGGTGGAGTAGAGG  SULF2 ATGGCACCCCCTGGCCTGCCACTAT CATAGACTTGCCCTTCACCAGCCC  SPRY4 CCCCGGCTTCAGGATTTA CTGCAAACCGCTCAATACAG  ETV4 GATGAAAGCCGGATACTTGGAC TTCGCGCAAGCTCCCATTT  TRIB3 AAGCGGTTGGAGTTGGATGAC CACGATCTGGAGCAGTAGGTG  COX2 AGGAGGTCTTTGGTCTGGTG TAGCCTGCTTGTCTGGAACA  AKR1C3 GGATTTGGCACCTATGCACCTC CTATATGGCGGAACCCAGCTTCTA  PLB1 GCATACACCAAGGAGGCTTTG GAGTGGATAGCAAGGAAGTGTCAC  GPX3 CCAACCACACTATCTACCC ACACAATCACGCATACCT  CYP4F3 GCTTTGACAGCCATTGCCA CATCTGTGAAGTCGTGCACCA  GGT1 GTGTTCTGCCGGGATAGAAA CAGGTCCTCAGCTGTCACAA  FOXA1 AGGAACTGTGAAGATGGAAGG ATGTTGCCGCTCGTAGTC  FOXA2 GGAGCAGCTACTATGCAGAGC CGTGTTCATGCCGTTCATCC  TFAP2C CGACATGGCACACCAGAT GGAAATAGGACCTTTGCGAATAAC  FOS CCAGTCAAGAGCATCAGCAA AAGTAGTGCAGCCCGGAGTA  GAPDH CCTCAAGATCATCAGCAAT CCATCCACAGTCTTCTGGGT |

**Supplementary Table S4. ChIP-qPCR primers**

| Name Forward (5’to3’) Reverse(5’to3’) |
| --- |
| Pro#1 CAAGTAGCACCAAGCCCTCC ACTCCACCTCCGCTCCAA  CUT#1 AGCAGTTAGGTCCACATTC TCTCCCTTGTATCCTTGTAT  CUT#2 CCCCTGCTGGACTGATAC CCTGGCTAACTTCTGCTC  CUT#3 GTTGCAGTGAGCCGAGAT GATGCTGGAATAAGGGTTT  CUT#4 GACTCCTGCGTGCTGACTG CGAGTATGAGGCCCGTGA  CUT#5 CTGAACTGAGCCAAACCT GGCACCTGATAAGCACCC  C1 CGAACTTCCGCCCTCCCA GCTGTATCCTGCCGCCACC  C2 TGCAGACCACAGCGCCCTCA GCTGCTCCTCAGACCTTCCTCC  F1 TCAGCCGCTTCAGGGAGT CGCGTGTTATGGGAGATG  F2 CCAAGATTATCCAAGGCAGTT CGGAGCATACCCACAAGG  P1 TGCTGTCATTTTCCTGTAATGCT GGAGCATGTGAGGGTGAGAT  P2 CCACTTCTTTTCTGGTGTGTGT CTCAGGGAGGAGCATGTGAG |

**Supplementary Table S5. Antibody**

| Name Co.,Ltd. Name Co.,Ltd. |
| --- |
| HA Proteintech Ubiquitin ABclonal  MMP9 Proteintech His ABclonal  β-catenin Proteintech CCT3 ABclonal  VEGF Proteintech TJP2 ABclonal  β-actin Proteintech GST ABclonal  RUVBL1 Proteintech Lyn CST  EIF3I Proteintech POLR2A CST  COX2 Proteintech P300 CST  H3 Proteintech Flag Cusabio  CTCF Proteintech RPS4X Cusabio  IgG Proteintech CKAP4 Cusabio  TRIB3 Abcam FOXA1 Thermo Fisher  H3K4me3 Active Motif H3K27ac Active Motif |


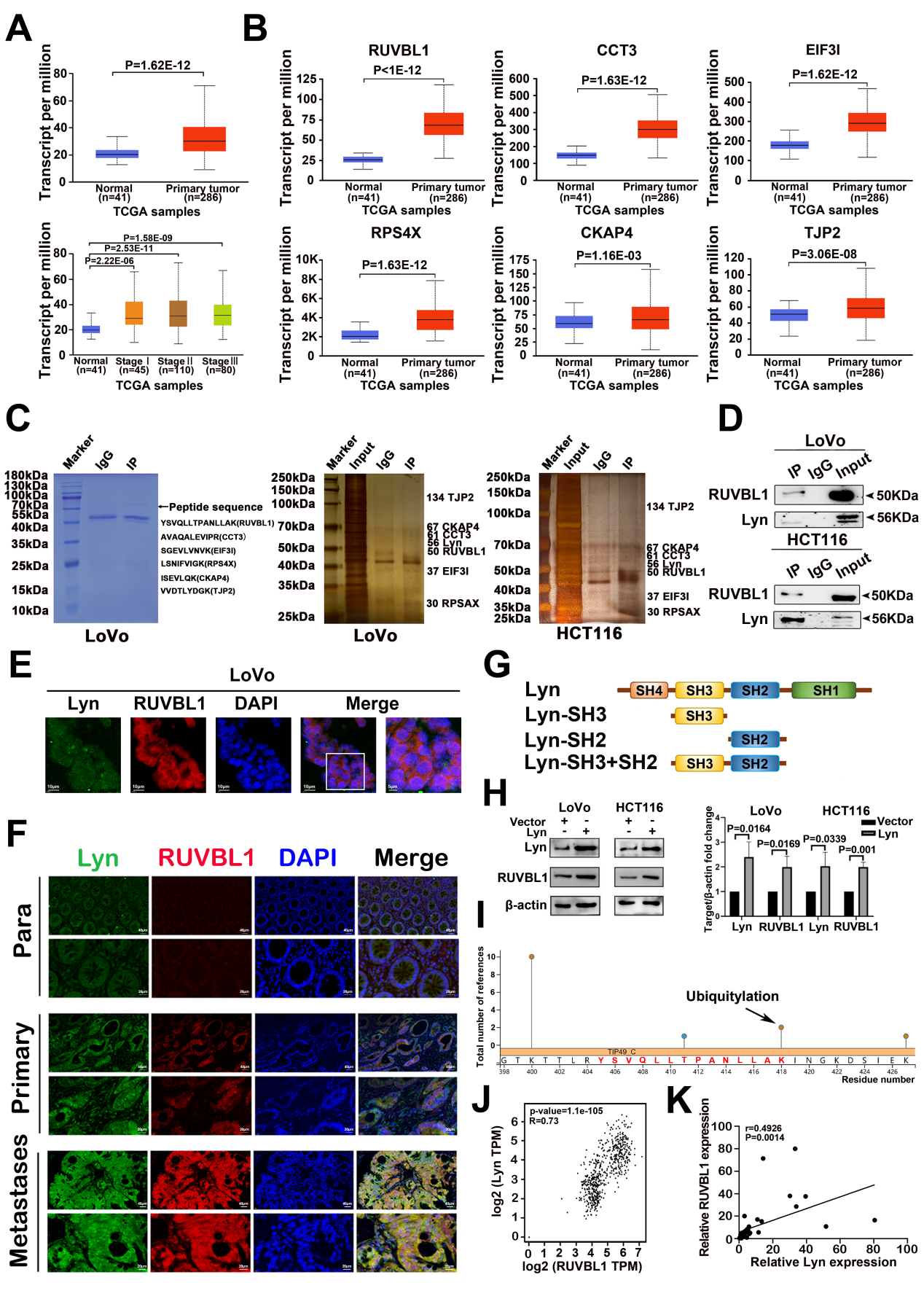


**Figure S1. Related to Figure 1.** (A) TCGA analysis of Lyn expression and staging in colon cancer tissues. (B) TCGA analysis of the expression of Lyn-bound proteins in colon cancer tissues. (C) Coomassie blue (left) and silver (right) stains. (D) An IP antibody targeting RUVBL1 was used to investigate its association with Lyn in colon cancer cells. (E) Localization of Lyn and RUVBL1 in LoVo cells was detected by IF. (F) Localization of Lyn and RUVBL1 in CRC and liver metastases was detected by IF. (G) Lyn truncated body diagram. (H) Western blot analysis of the effect of Lyn overexpression on RUVBL1 protein levels in colon cancer cells. (I) The phosphosite database displays modifications of RUVBL1 and Lyn binding sites. (J) The GEPIA database was used to analyze the correlation between Lyn and RUVBL1 expression in colon cancer tissues. (K) qRT‒PCR results were used to analyze the correlation between Lyn and RUVBL1 expression in the tissues of CRC patients. Data are presented as the means ± SDs. For (H), an unpaired t test was used.


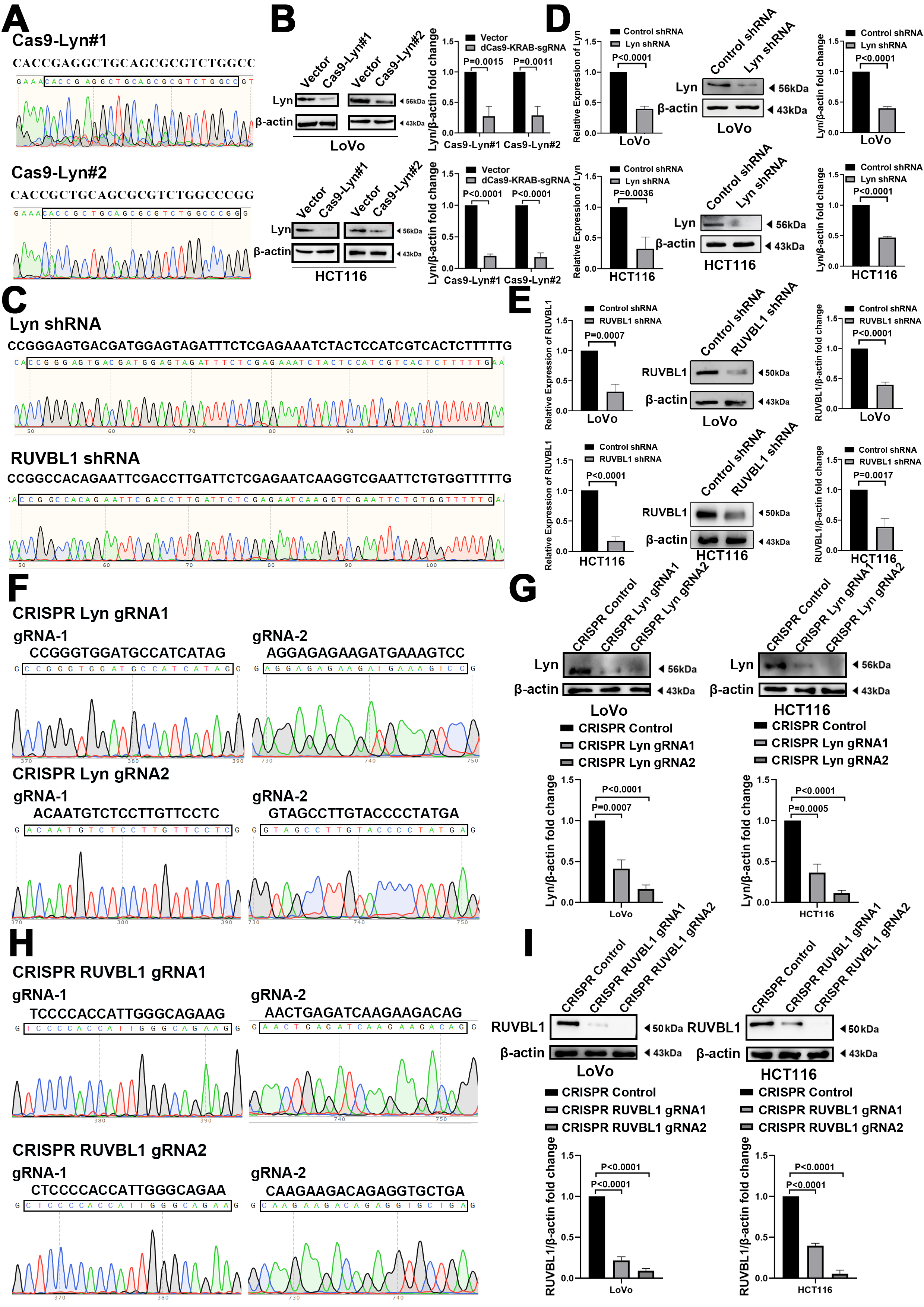


**Figure S2. Related to Figure 2.** (A) Sanger sequencing of the CRISPR/dCas9-KRAB plasmid. (B) Western blot analysis was performed to detect the knockout effect of CRISPR/dCas9-KRAB on Lyn expression. (C) Sanger sequencing of the Lyn and RUVBL1 recombinant lentiviral plasmids. (D) qRT‒PCR was used to detect the knockdown effect of Lyn lentivirus on Lyn mRNA expression (left). Western blot analysis of the knockdown effect of Lyn lentivirus on Lyn expression (right). (E) qRT‒PCR was used to detect the knockdown effect of RUVBL1 lentivirus on RUVBL1 mRNA expression (left). Western blot analysis of knockdown effect of RUVBL1 lentivirus on RUVBL1 expression (right). (F) Lyn CRISPR/Cas9 plasmid Sanger sequencing. (G) Western blot analysis of the effect of Lyn CRISPR/Cas9 knockout on Lyn expression. (H) Sanger sequencing of the RUVBL1 CRISPR/Cas9 plasmid. (I) Western blot analysis of the knockout effect of RUVBL1 CRISPR/Cas9 on RUVBL1 expression. Data are presented as the means ± SDs. For (B), (D, E), (G), and (I), unpaired t tests were performed.


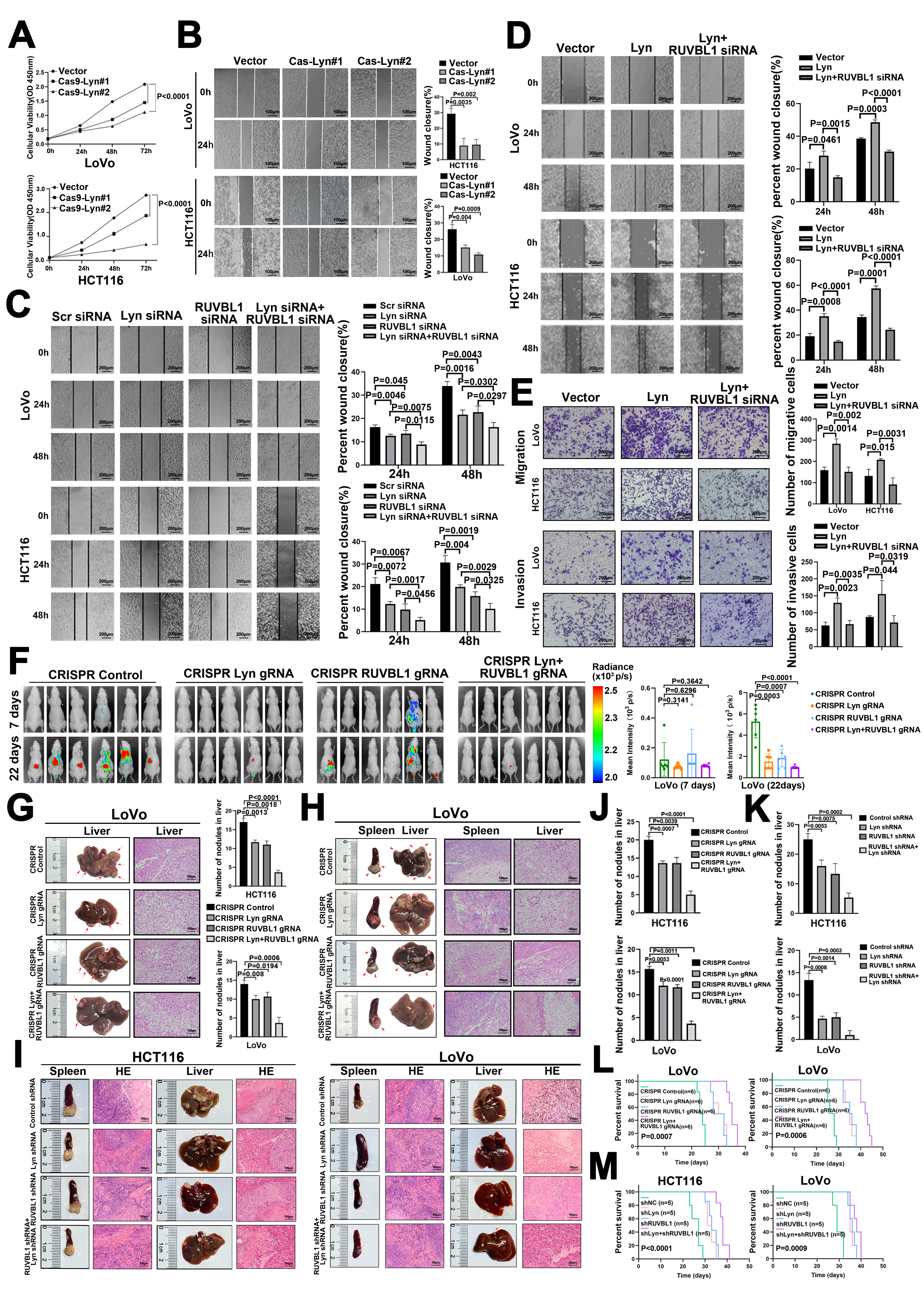


**Figure S3. Related to Figure 2.** (A) CCK8 assay to detect the effect of knocking out Lyn on the proliferation capacity of colon cancer cells. (B) Scratch assay to detect the effect of Lyn knockout on colon cancer cells migration. (C) The effect of the Lyn/RUVBL1 complex on colon cancer cells migration was detected via a scratch assay. (D) The effect of RUVBL1 knockdown on colon cancer cells migration after Lyn overexpression was detected via a scratch assay. (E) Effects of RUVBL1 knockdown on colon cancer cell migration and invasion after Lyn overexpression were detected via a transwell assay. (F) Bioluminescence imaging was used to detect fluorescence intensity and quantify fluorescence in a liver metastasis model of CRC in nude mice (n=6). (G) Liver tumor formation and HE staining of nude mouse livers after CRISPR/Cas9 knockout of Lyn and RUVBL1 in LoVo cells. (H) Tumor formation and HE staining of the spleen and liver of nude mice inoculated with CRISPR/Cas9 to knock out the LoVo cells of Lyn and RUVBL1. (I) Tumor formation and HE staining of the spleen and liver of nude mice inoculated with lentivirus after Lyn and RUVBL1 were knocked out in colon cancer cells. (J) Number of liver micrometastases after CRISPR/Cas9 knockout of Lyn and RUVBL1 in colon cancer cells in the nude mouse spleen. (K) Number of liver micrometastases after lentivirus knockout of Lyn and RUVBL1 in colon cancer cells in the spleens of nude mice. (L) Survival analysis of nude mice after liver (left) and spleen (right) CRISPR/Cas9 knockout of Lyn and RUVBL1 in LoVo cells. (M) Survival analysis of a nude mouse spleen inoculated with lentivirus knockdown of Lyn and RUVBL1 colon cancer cells. Data are presented as the means ± SDs. For (A), two-way ANOVA was used. For (B–G) and (J, K), an unpaired t test was used. For (L, M), the log-rank test was used.


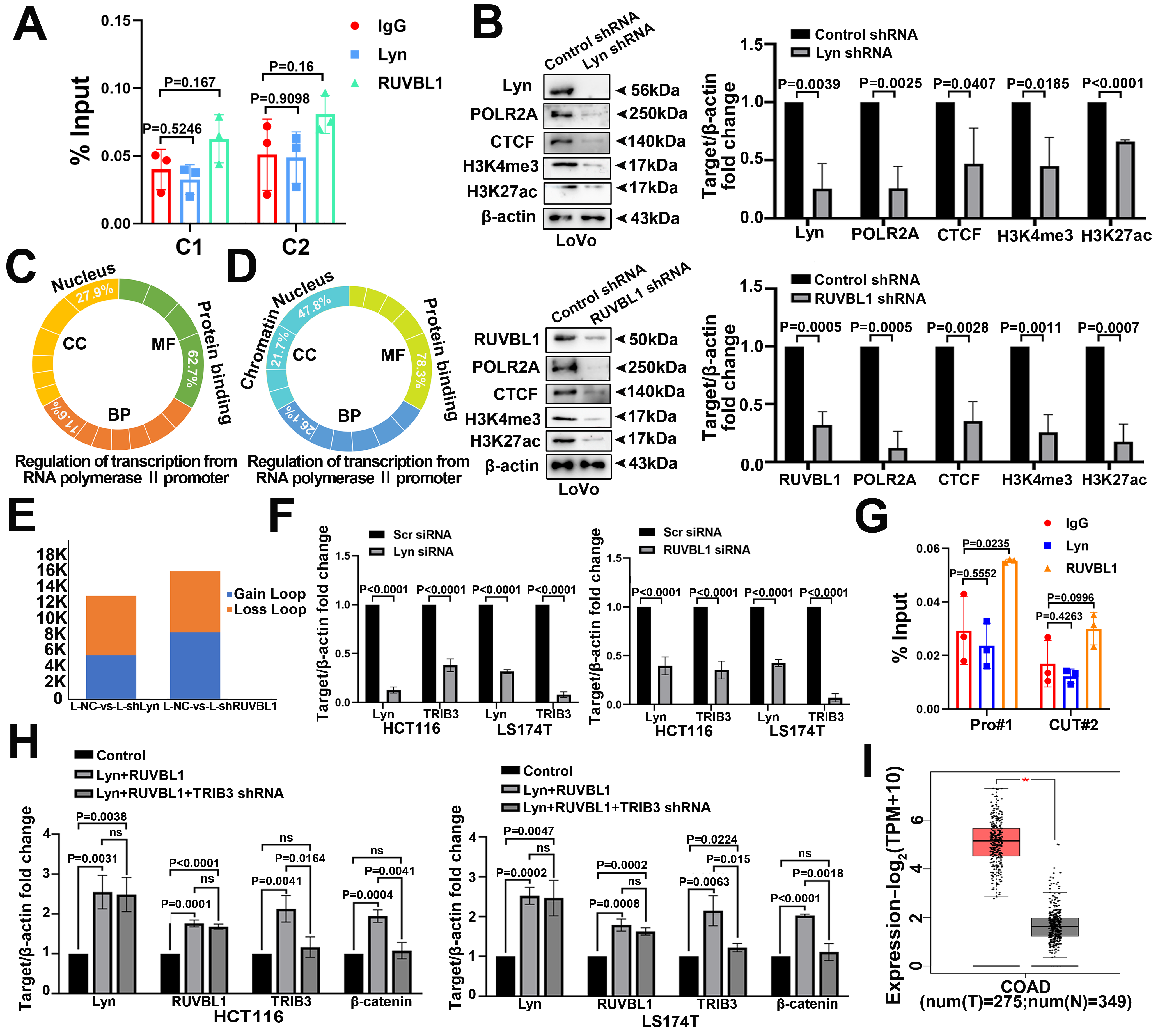


**Figure S4. Related to Figure 4.** (A) The combination of Lyn/RUVBL1 and CTNNB1 was analyzed via ChIP‒qPCR. (B) Western blot detection of chromatin remodeling-related protein expression after Lyn and RUVBL1 were knocked down. (C) GO analysis of ATAC-seq- and RNA-seq-downregulated genes in Lyn. (D) GO analysis of genes downregulated from Lyn and RUVBL1 identified via RNA-seq. (E) HiCuT difference loop analysis. (F) Grayscale analysis of TRIB3 after the knockdown of Lyn and RUVBL1. (G) The combination of Lyn/RUVBL1 and TRIB3 was analyzed via ChIP‒qPCR. (H) Grayscale analysis of β-catenin after the overexpression of Lyn/RUVBL1 knocked down TRIB3. (I) The expression of TRIB3 in colon cancer tissues was analyzed via TCGA. Data are presented as the means ± SDs. For (A, B), and (F–H), an unpaired t test was used.


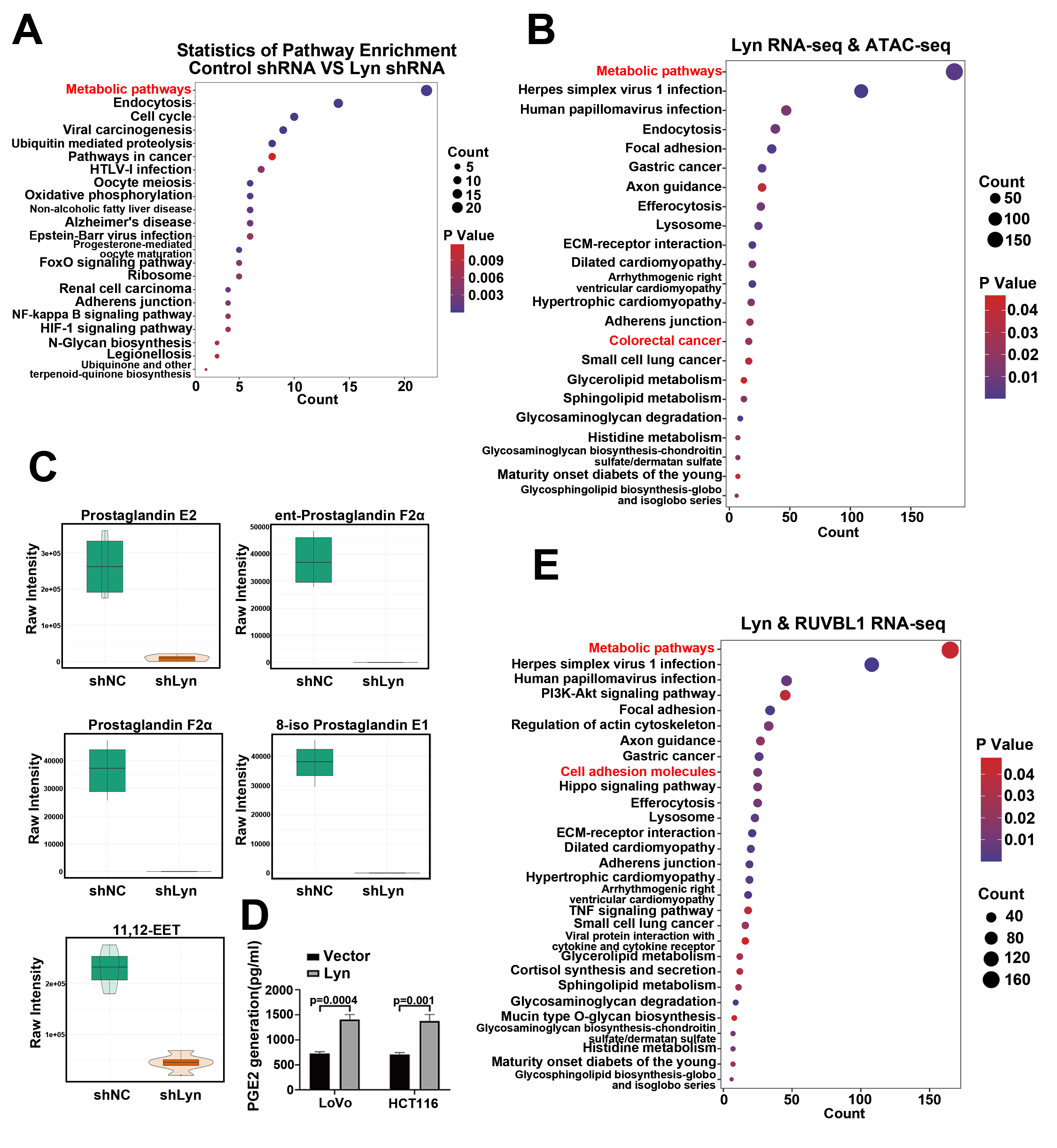


**Figure S****5. Related to Figure 5.** (A) KEGG analysis of ATAC-seq differentially expressed genes in LoVo cells after Lyn was knocked down. (B) KEGG enrichment analysis of differentially expressed genes after combined ATAC-seq and RNA-seq analysis using Lyn. (C) Violin diagram of the expression of molecules related to AA metabolism via metabolomics. (D) The expression level of PGE2 after the overexpression of Lyn was detected via ELISA. (E) KEGG enrichment analysis of differentially expressed genes after Lyn and RUVBL1 combined with RNA-seq analysis. Data are presented as the means ± SDs. For (D), an unpaired t test was used.


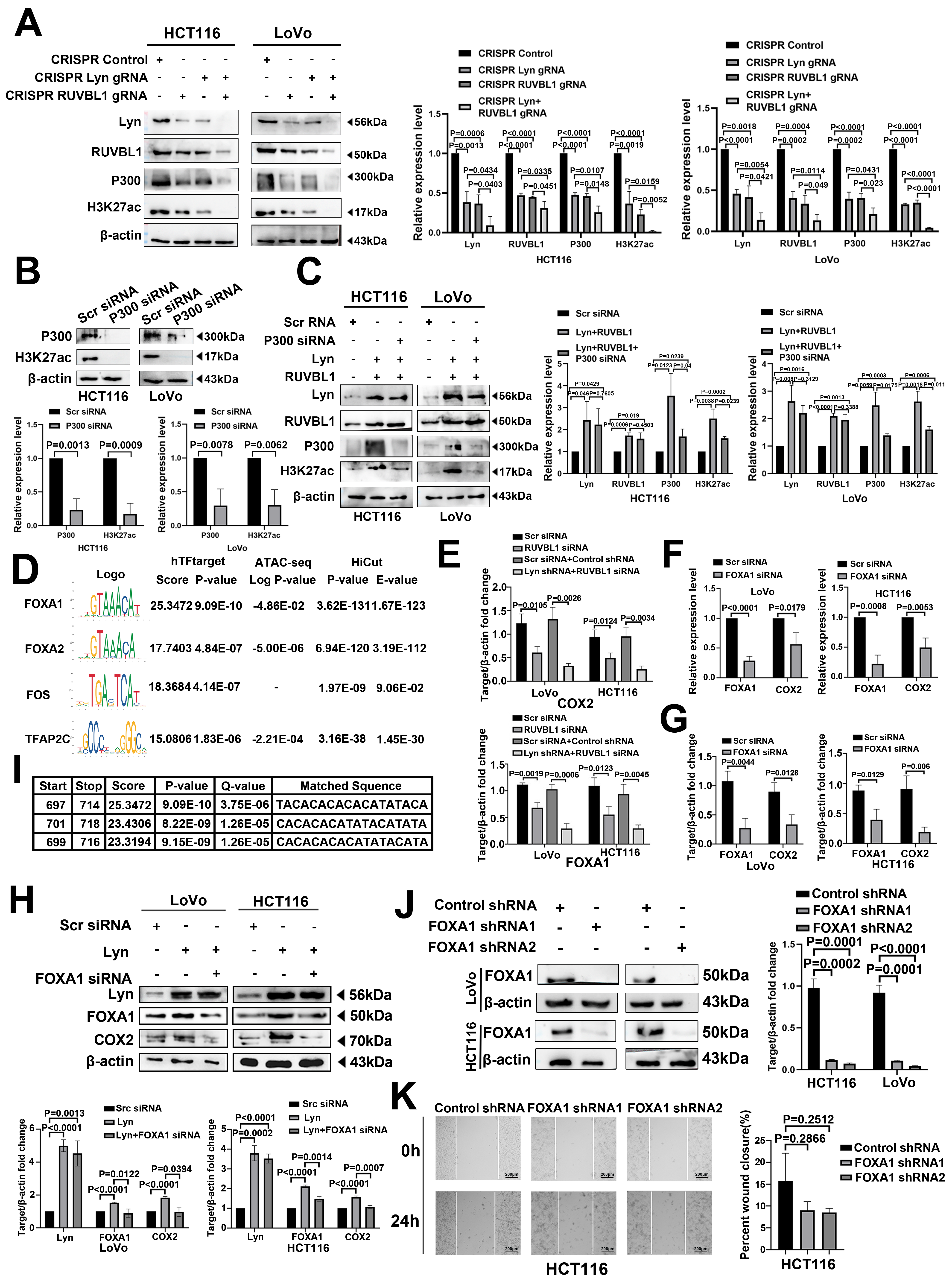


**Figure S****6. Related to Figure 6.** (A) Western blot was performed to detect the expression of P300 and H3K27ac after Lyn/RUVBL1 was knocked out. (B) Western blot analysis of H3K27ac expression after P300 knockdown. (C) Western blot detection of H3K27ac expression after P300 knockdown of Lyn/RUVBL1 overexpression. (D) Motif analysis of hTFtarget scoring in conjunction with Lyn ATAC-seq and HiCut. (E) Grayscale analysis of the effects of the Lyn/RUVBL1 complex on COX2 and FOXA1 protein levels in colon cancer cell lines by Western blotting. (F) qRT‒PCR was used to detect the effect of FOXA1 knockdown on COX2 mRNA expression in colon cancer cells. (G) Grayscale analysis of the effect of FOXA1 knockdown on COX2 protein levels in colon cancer cells. (H) Western blot analysis of the effect of FOXA1 knockdown on COX2 after the overexpression of Lyn in colon cancer cells. (I) hTFtarget prediction of the binding site of FOXA1 and COX2. (J) Western blot analysis of the knockdown effect of FOXA1 lentivirus on FOXA1 protein expression in colon cancer cells. (K) The effect of FOXA1 knockdown on HCT116 migration was detected via a scratch assay. Data are presented as the means ± SDs. For (A–C), (E–H), and (J, K), unpaired t tests were performed.


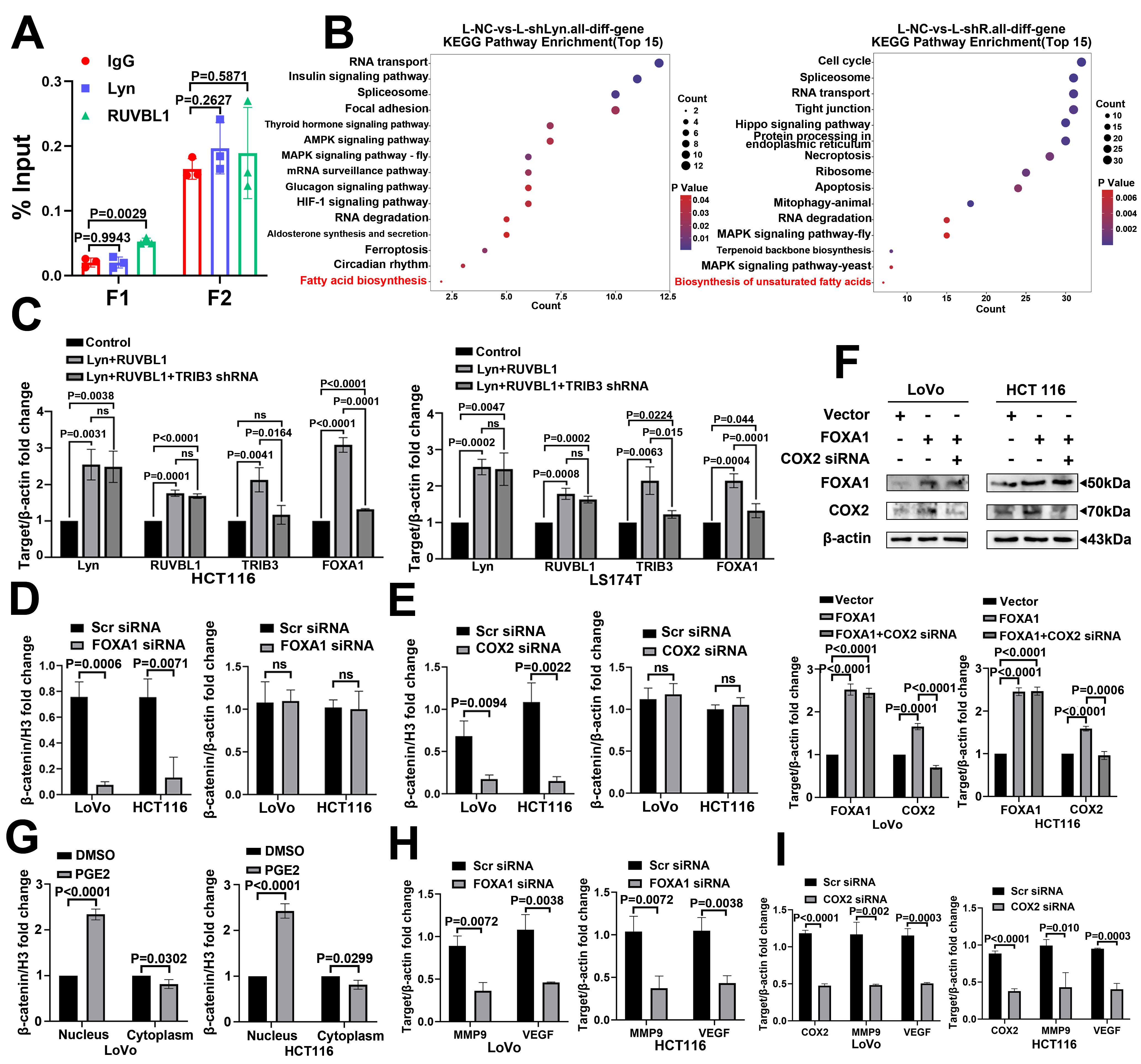


**Figure S7. Related to Figure 7.** (A) The binding of Lyn/RUVBL1 to FOXA1 was studied by ChIP‒qPCR. (B) KEGG pathway analysis of the HiCut differential genes. (C) Grayscale analysis after the overexpression of Lyn/RUVBL1 and knocked down TRIB3. (D) Grayscale analysis of β-catenin detected after FOXA1 was knocked down. (E) Gray analysis of β-catenin detected after COX2 was knocked own. (F) Western blot detection of overexpressed FOXA1 knocked down COX2 expression. (G) Grayscale analysis of the effect of PGE2 on β-catenin. (H) Grayscale analysis of the effects of FOXA1 knockdown on the expressions of MMP9 and VEGF in colon cancer cells. (I) Grayscale analysis of the effects of COX2 knockdown on MMP9 and VEGF expression in colon cancer cells. Data are presented as the means ± SDs. For (A), (C–I), an unpaired t test was used.
